# Sphingolipid desaturase DEGS1 is essential for mitochondria-associated membrane integrity

**DOI:** 10.1172/JCI162957

**Published:** 2023-05-15

**Authors:** Laura Planas-Serra, Nathalie Launay, Leire Goicoechea, Bénédicte Heron, Cristina Jou, Natalia Juliá-Palacios, Montserrat Ruiz, Stéphane Fourcade, Carlos Casasnovas, Carolina De La Torre, Antoinette Gelot, Maria Marsal, Pablo Loza-Alvarez, Àngels García-Cazorla, Ali Fatemi, Isidre Ferrer, Manel Portero-Otin, Estela Area-Gómez, Aurora Pujol

**Affiliations:** 1 Neurometabolic Diseases Laboratory, Bellvitge Biomedical Research Institute (IDIBELL), L’Hospitalet de Llobregat, Barcelona, Catalonia, Spain.; 2 Centre for Biomedical Research on Rare Diseases (CIBERER), Instituto de Salud Carlos III, Madrid, Spain.; 3 Department of Paediatric Neurology, Reference Centre for Neurogenetic Diseases, Armand Trousseau–La Roche Guyon University Hospital, and I2-D2 Federation, Sorbonne-Université, Paris, France.; 4 Neurometabolic Unit and Synaptic Metabolism Lab, Neurology and Pathology Department, Institut Pediàtric de Recerca, Hospital Sant Joan de Déu, and MetabERN, Barcelona, Catalonia, Spain.; 5 Neuromuscular Unit, Neurology Department, Hospital Universitari de Bellvitge, Universitat de Barcelona, L’Hospitalet de Llobregat, Barcelona, Catalonia, Spain.; 6 Josep Carreras Leukaemia Research Institute Barcelona, Catalonia, Spain.; 7 Armand Trousseau–La Roche Guyon University Hospital, Sorbonne-Université, Paris, France.; 8 ICFO–Institut de Ciències Fotòniques, The Barcelona Institute of Science and Technology, Castelldefels, Barcelona, Catalonia, Spain.; 9 Departments of Neurology and Pediatrics, The Kennedy Krieger Institute, and Johns Hopkins University School of Medicine, Baltimore, Maryland, USA.; 10 Department of Pathology and Experimental Therapeutics, University of Barcelona, L’Hospitalet de Llobregat, Barcelona, Catalonia, Spain.; 11 Network Centre of Biomedical Research of Neurodegenerative Diseases (CIBERNED), Institute of Health Carlos III, L’Hospitalet de Llobregat, Barcelona, Catalonia, Spain.; 12 Departament de Medicina Experimental, Universitat de Lleida–Institut de Recerca Biomedica de Lleida, Lleida, Catalonia, Spain.; 13 Department of Neurology, Columbia University Medical Center, New York, New York, USA.; 14 Centro de Investigaciones Biológicas “Margarita Salas,” Madrid, Spain.; 15 Catalan Institution of Research and Advanced Studies (ICREA), Barcelona, Catalonia, Spain.

**Keywords:** Metabolism, Neuroscience, Bioenergetics, Demyelinating disorders, Lipid rafts

## Abstract

Sphingolipids function as membrane constituents and signaling molecules, with crucial roles in human diseases, from neurodevelopmental disorders to cancer, best exemplified in the inborn errors of sphingolipid metabolism in lysosomes. The dihydroceramide desaturase Δ4-dihydroceramide desaturase 1 (DEGS1) acts in the last step of a sector of the sphingolipid pathway, de novo ceramide biosynthesis. Defects in DEGS1 cause the recently described hypomyelinating leukodystrophy-18 (HLD18) (OMIM #618404). Here, we reveal that DEGS1 is a mitochondria-associated endoplasmic reticulum membrane–resident (MAM-resident) enzyme, refining previous reports locating DEGS1 at the endoplasmic reticulum only. Using patient fibroblasts, multiomics, and enzymatic assays, we show that DEGS1 deficiency disrupts the main core functions of the MAM: (a) mitochondrial dynamics, with a hyperfused mitochondrial network associated with decreased activation of dynamin-related protein 1; (b) cholesterol metabolism, with impaired sterol *O*-acyltransferase activity and decreased cholesteryl esters; (c) phospholipid metabolism, with increased phosphatidic acid and phosphatidylserine and decreased phosphatidylethanolamine; and (d) biogenesis of lipid droplets, with increased size and numbers. Moreover, we detected increased mitochondrial superoxide species production in fibroblasts and mitochondrial respiration impairment in patient muscle biopsy tissues. Our findings shed light on the pathophysiology of HLD18 and broaden our understanding of the role of sphingolipid metabolism in MAM function.

## Introduction

Sphingolipids (SLs) are fundamental components of cellular membranes and bioactive signaling molecules that are constituted by a sphingoid base backbone coupled to a fatty acyl chain. Ceramide (Cer) serves as the central building block for more complex SL species. Generation of Cer mainly occurs via 3 pathways: (a) the de novo pathway, which uses palmitoyl-CoA and serine as its precursors, (b) the sphingomyelinase pathway, which converts sphingomyelin (SM) into Cer bidirectionally, and (c) the salvage pathway, which converts complex SLs into Cer (1, 2) (see Supplemental Figure 1; supplemental material available online with this article; https://doi.org/10.1172/JCI162957DS1).

The regulation of SL metabolism is critical in human physiology, as underscored by the numerous neurological and multisystemic inborn errors of SL metabolism that are caused by faulty enzymes of lysosomal SL degradation, such as Gaucher disease, Niemann-Pick disease, Krabbe disease, and metachromatic leukodystrophy (2, 3, 4). Recently, the advent of clinical genomics technologies and whole-exome and genome sequencing has uncovered the genetic causes of novel disease entities in the de novo synthesis pathway of SLs (see Supplemental Figure 1). Mutations in the coding gene of its first and rate-limiting enzyme, serine palmitoyltransferase long chain base subunit 1 (SPTLC1), cause a childhood-onset form of amyotrophic lateral sclerosis (5) and also a peripheral neuropathy (6, 7). Likewise, malfunction of Δ4-dihydroceramide desaturase 1 (DEGS1), which converts dihydroceramide (DhCer) into Cer (8) at the last step of this de novo pathway, causes severe hypomyelinating leukodystrophy-18 (HLD18) (OMIM #618404) (9, 10, 11). Beyond this rare disease, intriguing emerging evidence associates DEGS1 expression and aberrant levels of Cer and/or DhCer with the appearance of comorbidities in the context of obesity, including type 2 diabetes, insulin resistance, and cardiovascular diseases (12, 13, 14, 15, 16). However, the molecular mechanisms by which alterations in SL metabolism trigger human pathology are still under debate.

Although several enzymes of SL metabolism are located in lysosomes (17), numerous reports indicate that sphingomyelinase and Cer synthase activity, which are responsible for Cer biosynthesis, are significantly increased in specific domains of the ER found in close apposition to mitochondrial membranes, called mitochondria-associated ER membranes (MAMs) (18, 19, 20, 21). These ER structures are transient and dynamic lipid raft-like membranes that are pivotal for mitochondrial and cellular physiology. MAMs recruit multiple enzymes to orchestrate essential metabolic cascades, including phospholipid (PL) synthesis and transport, lipid droplet (LD) biogenesis, cholesterol esterification, cell metabolism of fatty acid esters, calcium homeostasis, and mitochondrial dynamics (22, 23, 24, 25), among other functions.

Here, we show that DEGS1 is not homogenously distributed in the ER as reported previously (10, 26, 27), but is enriched in MAM domains, expanding the known role of MAMs in Cer synthesis to the de novo branch of the pathway. Furthermore, we report that DEGS1 deficiency induces substantial alterations in lipid metabolism, the disruption of mitochondrial dynamics, and bioenergetics failure, broadening our understanding of this enzyme in rare and complex metabolic diseases.

## Results

### Aberrant mitochondrial homeostasis in DEGS1 patient muscle biopsies.

**Figure 1:**
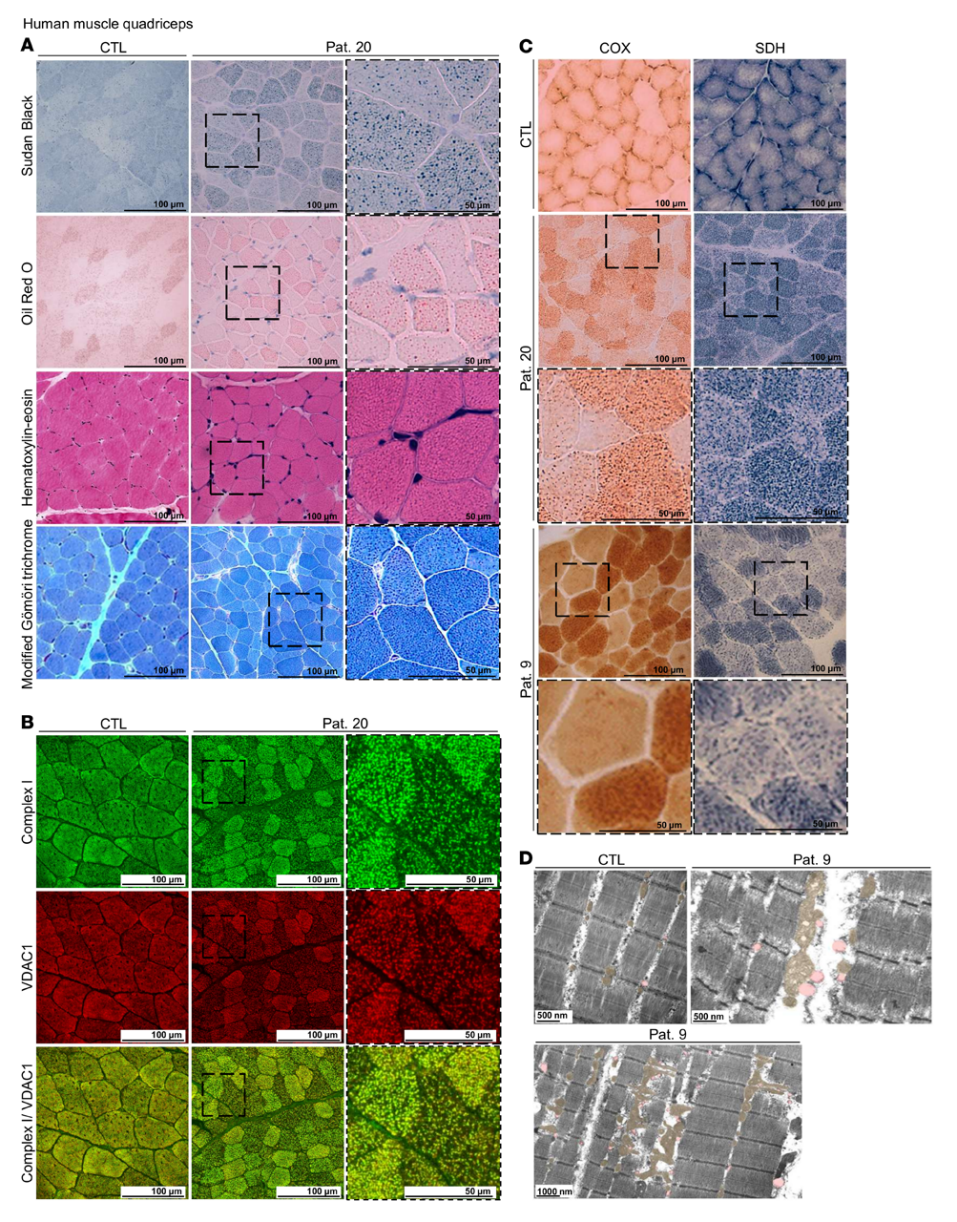
Mitochondrial damage in vivo. (**A**) Muscle histopathology. Sudan black and Oil Red O staining for neutral lipids, H&E staining for fiber size, and modified Gömöri trichrome staining for the endomysial connective tissue of the quadriceps muscle biopsy from Pat. 20 and a child who served as a control (CTL). (**B**) Immunofluorescence for complex I and VDAC1 markers in the quadriceps muscle biopsy from Pat. 20 and a child who served as a control. (**C**) Skeletal muscle histochemistry. COX and SDH staining of quadriceps muscle biopsies from Pat. 9 and Pat. 20 and a child who served as a control. (**D**) TEM of the quadriceps muscle biopsy in the longitudinal plane from Pat. 9 and a child who served as a control. Mitochondria and LDs are highlighted in orange and pink, respectively.

The clinical presentations of several *DEGS1* patients evaluated here (Supplemental Table 1) were compatible with those with a primary mitochondrial disorder, which prompted some centers (patients 9 [Pat. 9] and Pat. 20) to include muscle biopsies during the diagnostic workup prior to the establishment of a molecular diagnosis by whole-exome sequencing. For instance, histological and histochemical analysis of the quadriceps muscle biopsy from Pat. 20 revealed moderate variability in fiber size, with a population of hypotrophic fibers by H&E staining. Furthermore, there was an increase in myofibrillar lipid content, with larger LDs in both type I and II fibers, as revealed by Sudan black and Oil Red O staining (Figure 1A). No increase in the endomysial connective tissue, ragged red fibers, and subsarcolemmal accumulations of mitochondria were detected by modified Gömöri trichrome stain, although granular structures inside the fibers were observed (Figure 1A). Moreover, our immunofluorescence data from Pat. 20 showed that these granular and larger structures were mitochondria, as they costained with complex I and the mitochondrial voltage-dependent anion channel 1 (VDAC1) (Figure 1B).

Moreover, muscle tissues from both Pat. 9 and Pat. 20 presented with a population of fibers in mosaic with pale cytochrome *c* oxidase (COX) staining, showing an immature pattern in the absence of mitochondrial proliferation (Figure 1C). These findings suggest a loss of enzymatic activity for complex IV. In the same fibers, we observed a mosaic pattern with pale succinate dehydrogenase (SDH) staining (Figure 1C) indicating loss of complex II enzymatic activity. In line with the complex I and VDAC1 data for Pat. 20, SDH and COX stains revealed a coarse granular appearance compared with that for controls (Figure 1C).

The quadriceps muscle biopsy of Pat. 9 was further studied by transmission electron microscopy (TEM) and displayed increased numbers of LDs in close apposition to mitochondria (Figure 1D). Furthermore, in most muscle fibers, mitochondria showed alterations in size, length, and shape, with higher transverse diameter (500 to 800 nanometers) in comparison with controls (400 nanometers) and length up to 3 microns (Figure 1D and Supplemental Figure 2A). Additionally, some mitochondria presented irregular shapes, such as triangular, angular, branched, and alternation of strangulation and dilation areas (Figure 1D). Moreover, some mitochondrial cristae were abnormally profuse and densely packed (Figure 1D). These findings may suggest an impairment in the regulation of mitochondrial dynamics.

To further characterize these mitochondrial abnormalities, we measured the oxygen consumption rate (OCR) in isolated mitochondria from the muscle tissue from Pat. 9 and in total fibroblasts from Pat.9 and Pat.20, confirming a defect in the oxidative phosphorylation system (OXPHOS) complexes (Table 1 and Supplemental Table 2). In isolated mitochondria from muscle tissue, in the presence of either pyruvate or succinate as carbon sources, the OCR was significantly decreased compared with that of controls, as was the activity of all OXPHOS complexes. In addition, citrate synthase activity was decreased, and lactate dehydrogenase activity was increased in muscle from Pat. 9 (Table 1). The respiratory chain data indicated a very low mitochondrial activity with decreased mitochondrial mass in *DEGS1* patient muscle and fibroblasts, which is a similar finding to that of genetic disorders of complex subunits encoded by mitochondrial DNA (mtDNA). Together, these data indicate that dysfunction of DEGS1 affects mitochondrial morphology and respiration in quadriceps muscle and fibroblasts, inducing important bioenergetic defects.

### Mitochondrial aberrant morphology in DEGS1 patient fibroblasts.

**Figure 2:**
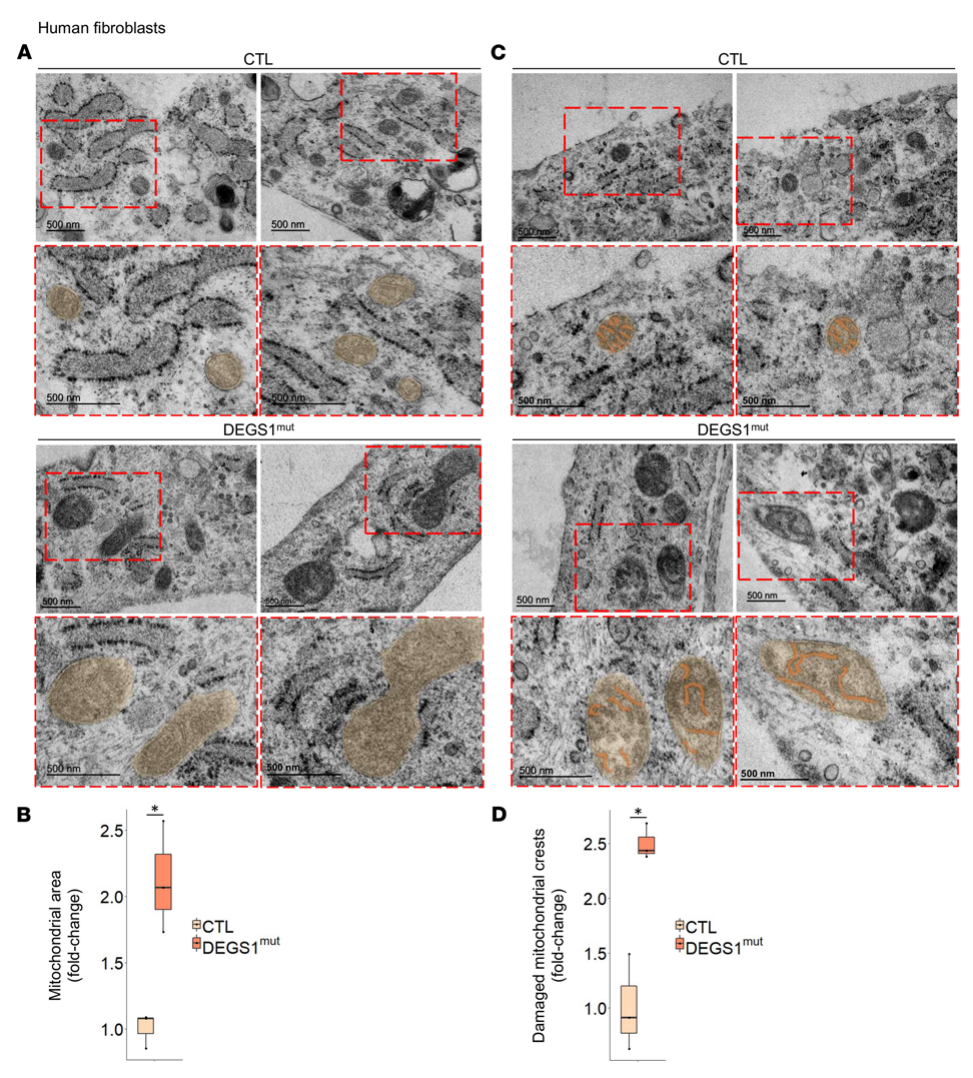
Mitochondrial aberrant morphology in *DEGS1* patient fibroblasts. Mitochondrial (**A**) area and (**C**) crests morphology assessment by TEM and their quantification (**B** and **D**), respectively. *DEGS1* patient (*n* = 3) and control (*n* = 3) fibroblasts. Data are presented as box-and-whisker plots (median, interquartile interval, minimum, maximum). *P < 0.05, 2-tailed Student’s *t* test.

We explored mitochondrial morphology in fibroblasts using TEM. The mitochondrial area was increased in *DEGS1* patient fibroblasts in comparison with those of controls (Figure 2, A and B). Moreover, we observed that the mitochondrial crests presented abnormal morphologies in *DEGS1* patient cells (Figure 2, C and D). Since OXPHOS complexes are found at the mitochondrial crests (28), the abnormal morphology of the crests may help explain the decreased activity observed in *DEGS1* patient muscle and fibroblasts.

### DEGS1 deficiency induces mitochondrial dynamics defects and increased mitochondrial ROS production.

**Figure 3:**
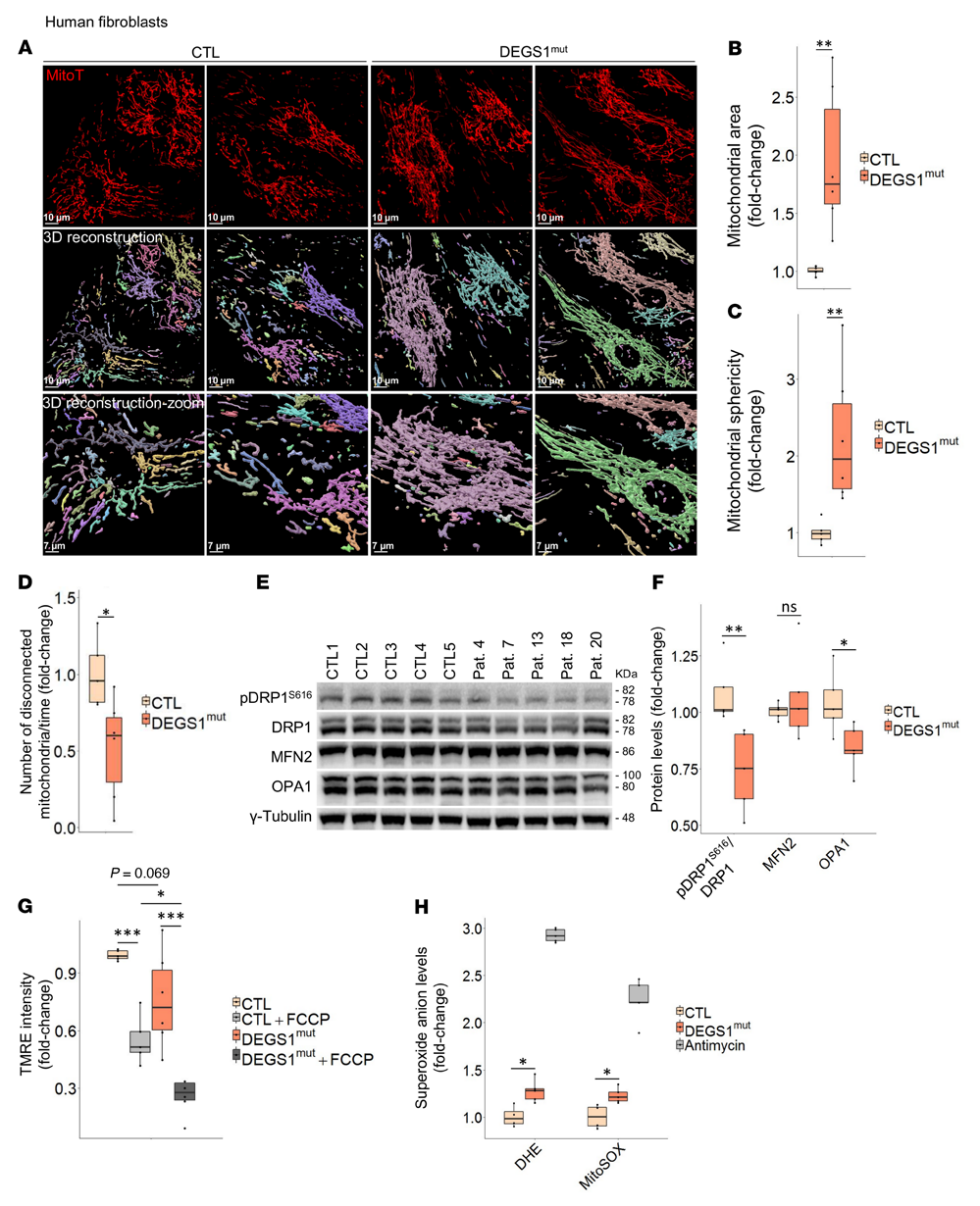
Mitochondrial dynamics and bioenergetics impairment in *DEGS1*patient fibroblasts. (**A**) Analysis of the mitochondrial morphology by MitoTracker (MitoT) staining in 4-hour live-cell imaging; disconnected mitochondria are each shown in different colors. Mitochondrial (**B**) area and (**C**) sphericity quantifications. (**D**) Quantification of numbers of disconnected mitochondria over time. *DEGS1* patient (*n* = 6) and control (*n* = 5) fibroblasts. (**E**) Western blot analysis of fusion and fission proteins and (**F**) their quantification. *DEGS1* patient (*n* = 5) and control (*n* = 5) fibroblasts. (**G**) Quantification of ΔΨm by measuring TMRE intensity. FCCP was used as a mitochondrial oxidative phosphorylation uncoupler. *DEGS1* patient (*n* = 6) and control (*n* = 5) fibroblasts. (**H**) Quantification of intracellular (DHE) and mitochondrial (MitoSOX) superoxide species production levels. The complex III inhibitor antimycin A (200 μM) was used for treatment for 1 hour as a positive control for ROS production. *DEGS1* patient (*n* = 5) and control (*n* = 4) fibroblasts. All experiments were done in triplicate. Data are represented as box-and-whisker plots (median, interquartile interval, minimum, maximum). *P < 0.05; **P < 0.01; ***P < 0.001, Wilcoxon’s test.

We next performed live-cell imaging studies in fibroblasts to obtain a 3D view of the mitochondrial morphology. Using MitoTracker as a fluorescence marker, we confirmed that mitochondria appeared hyperfused and exhibited increased mitochondrial area and sphericity in *DEGS1* patient fibroblasts compared with controls (Figure 3, A–C).

To investigate whether fusion and fission imbalances could underlie this hyperfused phenotype, we compared the numbers of disconnected mitochondria at the end of the live-cell imaging experiment (4 hours) to the ones at the beginning of it, using Imaris software. We observed that the numbers of disconnected mitochondria in *DEGS1* patient fibroblasts decreased, suggesting lesser fission events than in controls (Figure 3D and Supplemental Videos 1–4).

We then measured the levels of the main essential proteins involved in these fusion and fission processes: dynamin-related protein 1 (DRP1) and its phosphorylated form (pDRP1^S616^), mitofusin 2 (MFN2), and optic atrophy protein 1 (OPA1). DRP1 is a GTPase responsible for mitochondrial fission and mitochondrial membrane scission, and it requires phosphorylation at serine 616 to become active. Once phosphorylated, pDRP1^S616^ translocates to mitochondria, where it oligomerizes and binds to its adaptors at constriction sites, leading to mitochondrial fission (29). MFN2 and OPA1 are GTPases that mediate inner and outer mitochondrial membrane fusion, respectively (30, 31). However, a shift toward shorter OPA1 isoforms has been associated with increased mitochondrial fission (31). Consistent with the hyperfused phenotype, the ratio pDRP1^S616^/DRP1 was significantly decreased in *DEGS1* patient fibroblasts. Conversely, while we found a decrease in OPA1 levels, MNF2 levels were not significantly altered (Figure 3, E and F). We evaluated the mitochondrial protein levels using VDAC1 as a mitochondrial marker and observed no differences between *DEGS1* patient fibroblasts and controls (Supplemental Figure 2, B and C).

*DEGS1*-dependent morphological alterations are reflected in function defects. Mitochondrial membrane potential (ΔΨm) is a key indicator of mitochondrial activity and depends on the permeability of the membranes (32). The ΔΨm of fibroblasts treated with the uncoupler carbonyl cyanide 4-(trifluoromethoxy) phenylhydrazone (FCCP) was significantly decreased in *DEGS1* patients (Figure 3G).

We have recently reported that total intracellular ROS production was increased in *DEGS1* patient fibroblasts (9). Cellular sources of ROS production are varied, including the ER, mitochondria, peroxisome, xanthine oxidase, cyclo-oxygenases, cytochrome p450 enzymes, lipoxygenases, flavin-dependent demethylase, oxidases for polyamines and amino acids, and nitric oxide synthases that produce oxidants (33). Given the abnormal mitochondrial morphology, we compared the total superoxide species production in the cell (using dihydroethidium [DHE]) to the superoxide generated only at mitochondria (using MitoSOX), as previously described (34). Our results revealed that the mitochondrial compartment is a superoxide overproduction site in *DEGS1* patient fibroblasts (Figure 3H), although we cannot rule out other sources.

### DEGS1 is localized at the MAMs.

**Figure 4:**
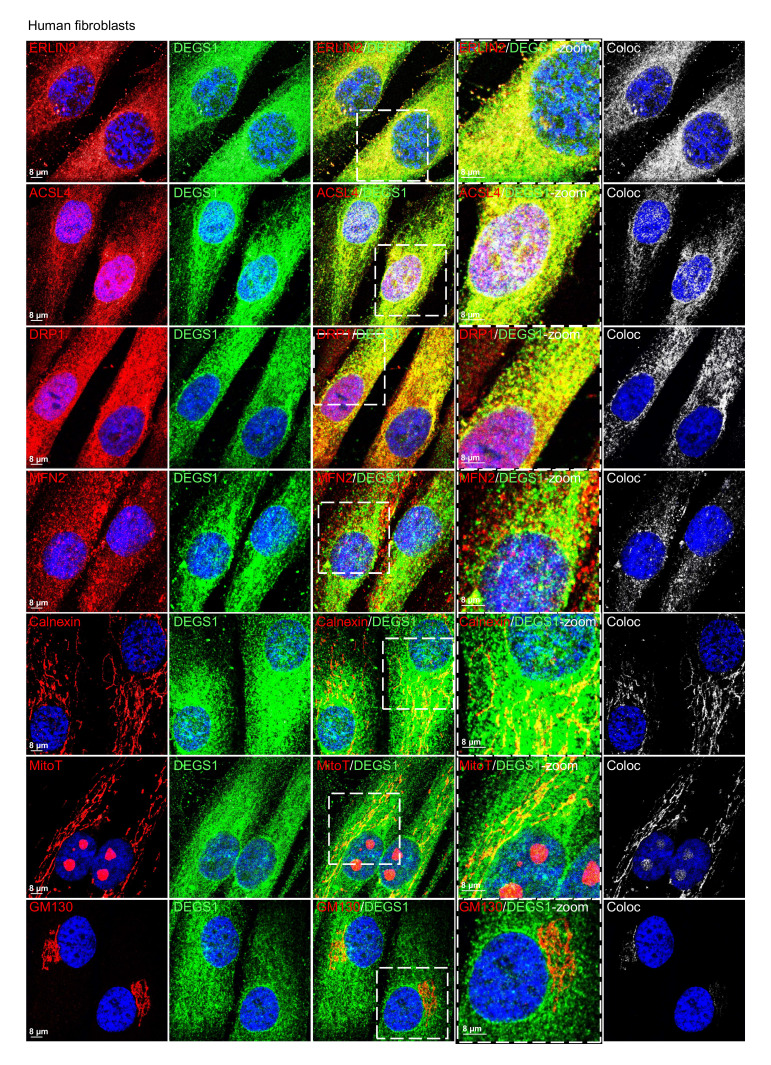
DEGS1 localizes at the MAM. Immunofluorescence analysis in control fibroblasts (*n* = 5). Colocalization between DEGS1 (in green) and ERLIN2, ACSL4, DRP1, and MFN2 (MAM-resident proteins), MitoTracker (mitochondria maker), calnexin (ER marker), and GM140 (Golgi marker) (all in red). Colocalization area (coloc) is shown in white.

In light of these data indicating the malfunction of mitochondrial respiration and dynamics, we set out to revisit the reported localization of DEGS1 at the ER (26, 27). We first explored the subcellular localization of DEGS1 in control fibroblasts. We performed immunofluorescence costaining with markers for specific cell compartments: ER (calnexin), mitochondria (MitoTracker), MAM (ERLIN2, ACSL4, DRP1 and MFN2), and Golgi apparatus (GM130). We found that DEGS1 was enriched in the ER areas next to mitochondria, colocalizing with MAM-resident proteins. In contrast, we detected less colocalization with MitoTracker or calnexin and essentially no colocalization with GM130 (Figure 4 and Supplemental Figure 3).

**Figure 5:**
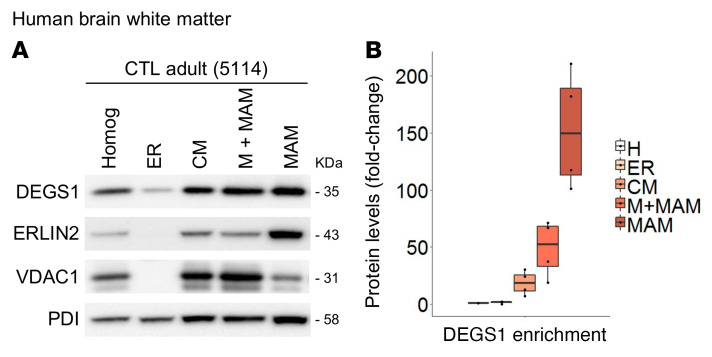
DEGS1 is detected in MAM-isolated fractions. (**A**) Western blot analysis of all recovered fractions during MAM collection from human brain white matter of control cases, adults (*n* = 2) and children (*n* = 2). Note that DEGS1 is more abundant in the MAM fraction than in the ER fraction (25 μg of protein per lane). (**B**) Representation of DEGS1 enrichment normalized to the total amount of protein in each fraction. Data are represented as box-and-whisker plots (median, interquartile interval, minimum, maximum). Homog, homogenate; CM, crude mitochondria; M+MAM, mitochondria attached to MAM.

To confirm these results in a tissue with high myelin content, we isolated subcellular fractions from human brain subcortical white matter of healthy individuals and analyzed them by Western blot using specific markers for each compartment. In agreement with our immunofluorescence colocalization data, DEGS1 was present in several cell compartments, although it was significantly enriched in the MAM fraction (Figure 5 and Supplemental Figure 4). Likewise, quantitative proteomics analysis of enriched MAM fractions from the spinal cord of WT mice revealed high levels of DEGS1 and known MAM-resident proteins such as ERLIN2 among others (Table 2), further supporting the contribution of SL homeostasis to the integrity of this dynamic domain.

As a lipid raft–enriched fraction, the MAM is a transient functional membrane domain formed by local increases in cholesterol (35). Therefore, we used an additional technique to more directly verify whether DEGS1 was located at the MAM. We incubated WT mouse embryonic fibroblasts (MEFs) with PhotoClick cholesterol, a cholesterol analog conjugated to a photoreactive alkyne that mimics native cholesterol. Using these cells, we next isolated MAM domains by subcellular fractionation and pulled down click cholesterol by conjugation to an azide-biotin tag followed by binding streptavidin beads, as described (36), and performed a proteomics analysis. Our results showed that several well-known MAM markers, such as ERLIN2 and ACLS4, were detected bound to cholesterol at MAM domains. Moreover, we were also able to detect DEGS1 bound to cholesterol in the MAM fractions, confirming our previous localization studies (Table 3).

### DEGS1 is required for MAM integrity and function.

**Figure 6:**
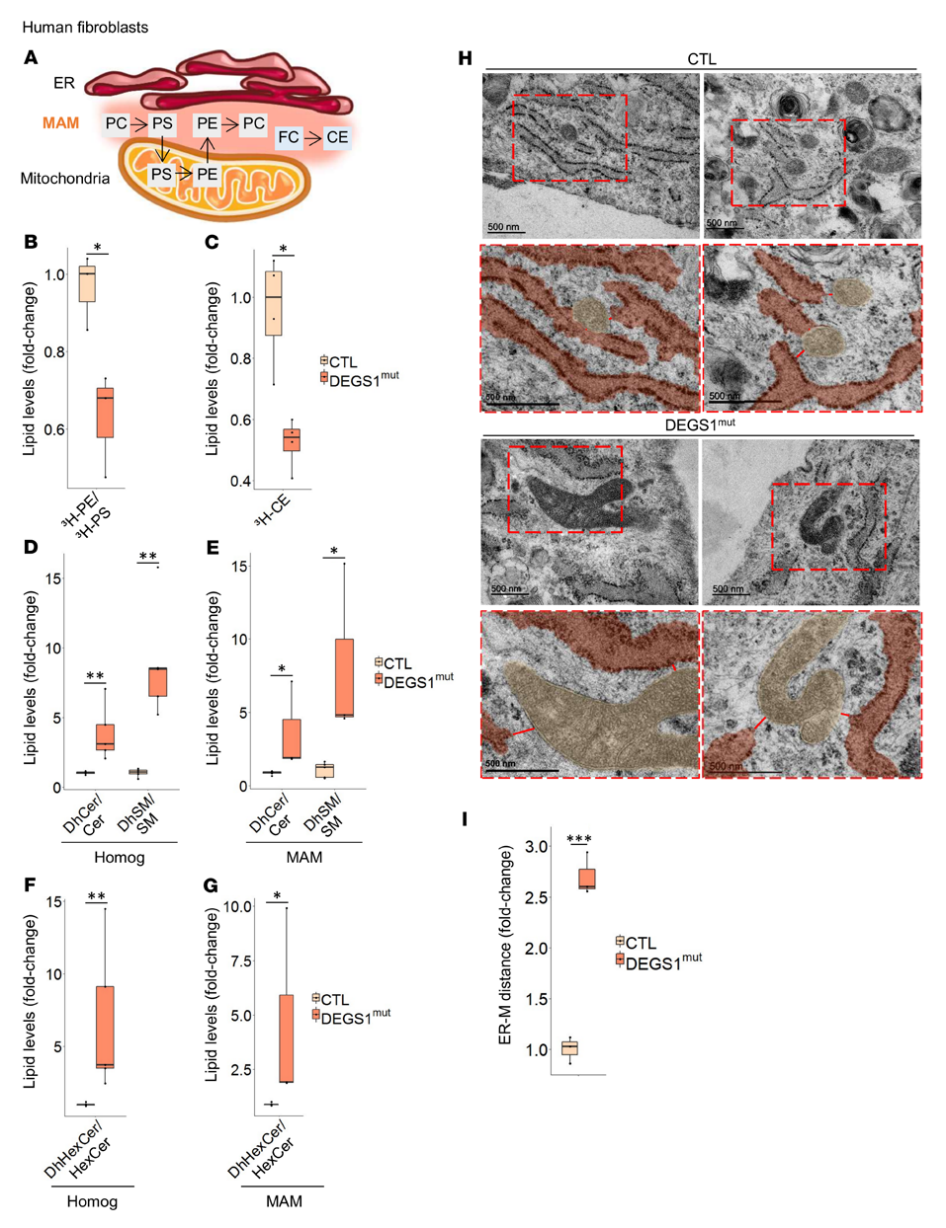
DEGS1 impairment leads to MAM disruption. (**A**) Schematic representation of PL synthesis and trafficking and cholesterol esterification at the MAM. (**B**) PL synthesis and trafficking assay. ^3^H-PS/^3^H-PE lipid ratio reflecting incorporation after 12 hours of ^3^H-serine into ^3^H-PS and ^3^H-PE in human fibroblasts from patients with *DEGS1* mutations (*n* = 3) and control individuals (*n* = 3). (**C**) SOAT1 activity assay. ^3^H-CE lipid levels reflecting the incorporation of ^3^H-cholesterol into ^3^H-CE after 6 hours in human fibroblasts from patients with *DEGS1* mutations (*n* = 4) and control individuals (*n* = 5). (**D** and **E**) DhCer/cer, DhSM/SM, and (**F** and **G**) DhHexCer/HexCer lipid levels in the total and MAM fractions from fibroblasts of patients with *DEGS1* mutations (*n* = 3–5) and control individuals (*n* = 4–5). (**H**) Distance between the ER and the mitochondria (M) in fibroblasts using TEM and its (**I**) quantification. *DEGS1* patient (*n* = 3) and control (*n* = 3) fibroblasts. Data are represented as box-and-whisker plots (median, interquartile interval, minimum, maximum). *P < 0.05; **P < 0.01, 2-tailed Student’s *t* test.

We next tested the potential functional impact of DEGS1 deficiency on MAM activities, focusing first on the synthesis of phosphatidylethanolamine (PE). This process involves the following steps: (a) phosphatidylserine (PS) synthesis by PS synthases 1 and 2 (PTDSS1/2) at the MAM, (b) its transport, and (c) its decarboxylation into PE by PS decarboxylase (PISD) in the mitochondria (37) (Figure 6A). For that, *DEGS1* patient and control fibroblasts were incubated in a medium containing ^3^H-serine, and after 12 hours, we measured the incorporation of the label into newly synthesized ^3^H-PS and ^3^H-PE. Notably, compared with control fibroblasts, all patient fibroblasts showed a decrease in the conversion of ^3^H-PS into ^3^H-PE (Figure 6B). This finding suggests a defect in MAM function and indicates impaired ER-mitochondria crosstalk in *DEGS1* patients. In agreement, the activity of sterol *O*-acyltransferase 1 (SOAT1, also known as ACAT1), a MAM-resident enzyme that catalyzes the conversion of free cholesterol (FC) to cholesteryl ester (CE) (20, 38) (Figure 6A), was significantly decreased in *DEGS1* patient fibroblasts (Figure 6C).

We and others previously reported that dysfunction of DEGS1 increases the DhCer/Cer ratio, and it also affects the ratios of other dihydrosphingolipids (dihydroSLs) in patient fibroblasts (9, 10, 11). To expand the molecular characterization of this phenotype, we performed a targeted lipidomics analysis of total fibroblasts and isolated MAMs from fibroblasts of *DEGS1* patients and controls. Our results indicated that DEGS1 impairment increased the levels of DhCer and dihydrosphingomyelin (DhSM) in both the total homogenate and the MAM fraction compared with their unsaturated forms, Cer and SM (Figure 6, D and E). Moreover, in *DEGS1* patient fibroblasts, the levels of dihydrohexosylceramide (DhHexCer) were increased compared with the levels of their unsaturated form hexosylceramide (HexCer) in both total homogenate and MAM-isolated membranes (Figure 6, F and G). Galactosylceramide (GalCer) is the main lipid constituent of myelin, which in addition to glucosylceramide (GluCer) are the 2 types of HexCer that are found in mammals. Thus, it appears that DEGS1 activity is not only necessary for the conversion of DhCer to Cer, but is also necessary for the conversion of most, if not all, of the dihydroSLs to their saturated forms. This aberrant lipidic composition likely drives the myelination defects observed in our patients (9).

We next performed TEM in *DEGS1* patient and control fibroblasts to assess the distance between the mitochondria and the ER, which usually ranges from 10 to 50 nm in this cell type (39). We observed that the mean distance between the 2 organelles was increased more than 2-fold in *DEGS1* patient cells (mean = 80.955 ± 6.293 nm) compared with controls (mean = 30.048 ± 3.923 nm) (Figure 6, H and I). This abnormally larger distance suggests that MAM formation may be physically hampered. Taken together, these data indicate that the impairment of DEGS1 induces remarkable alterations in the functional and structural connection between the ER and mitochondria.

### DEGS1 deficiency affects the homeostasis of LDs, neutral lipids, and PLs.

**Figure 7:**
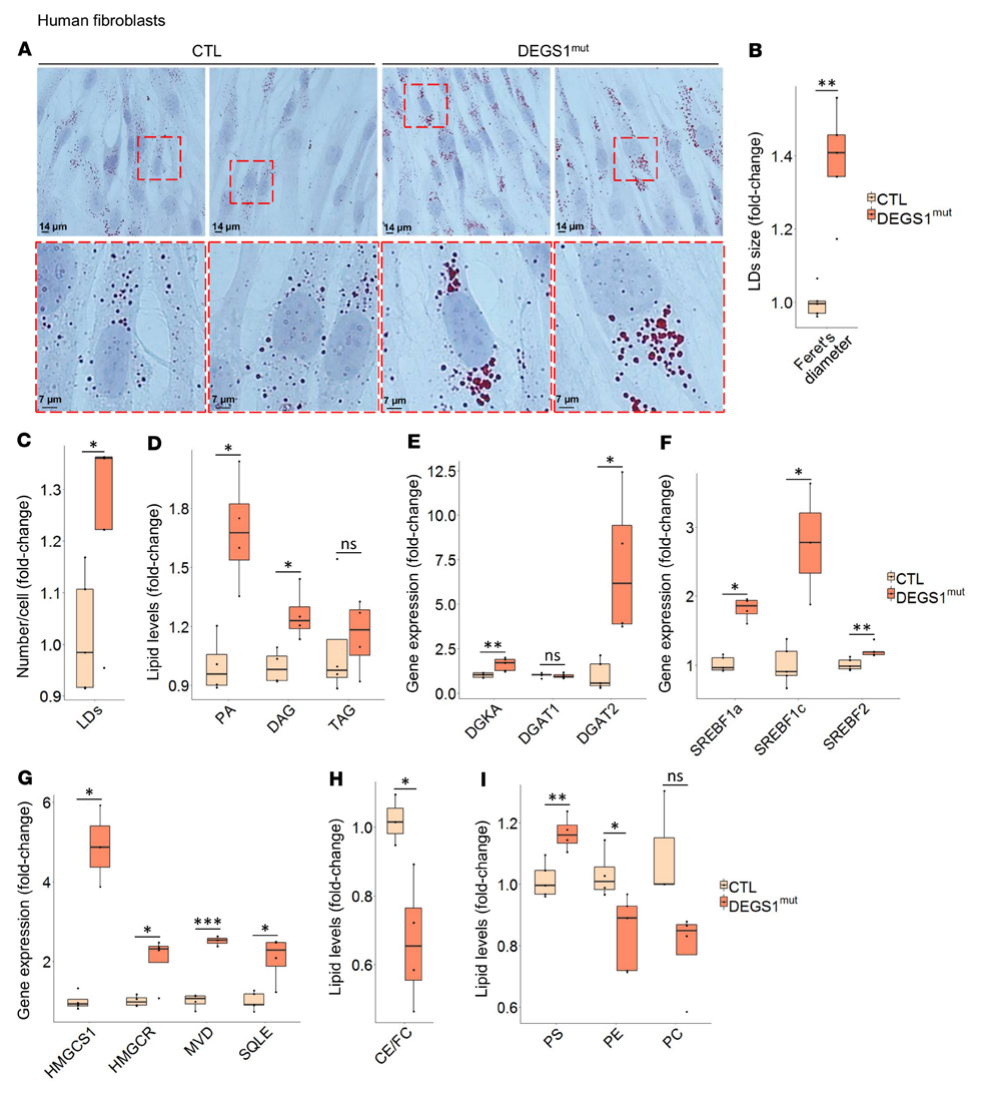
Neutral lipids and PA accumulation and their regulation. (**A**) Representative microscopy images of Oil Red O, an esterified cholesterol and LD marker, counterstained with hematoxylin as a nuclear marker, and its (**B**) Feret’s diameter and (**C**) number/cell quantification. *DEGS1* patient (*n* = 5) and control (*n* = 5) fibroblasts. (**D**) PA and glyceride levels. *DEGS1* patient (*n* = 4) and control (*n* = 4) fibroblasts. mRNA levels of (**E**) genes encoding key enzymes involved in the LD synthesis pathway *DGKA*, *DGAT1*, and *DGAT2*, and (**F**) the lipogenic gene master regulators *SREBF1a*, *SREBF1c*, and *SREBF2*. *DEGS1* patient (*n* = 4–5) and control (*n* = 5) fibroblasts. (**G**) mRNA levels of SREBF target genes involved in cholesterol synthesis: *HMGCS1*, *HMGCR*, *MVD*, and *SQLE*. *DEGS1* patient (*n* = 4) and control (*n* = 4) fibroblasts. (**H**) CE/FC lipid ratio and (**I**) PS, PE, and PC lipid levels in human fibroblasts from patients with *DEGS1* mutations (*n* = 4–5) and control individuals (*n* = 4–5). All experiments were done in triplicate. Data are presented as box-and-whisker plots (median, interquartile interval, minimum, maximum). *P < 0.05; **P < 0.01; ***P < 0.001, 2-tailed Student’s *t* test.

Alterations in the SL composition of the MAM result in significant changes in cellular lipid metabolism. In turn, these dysfunctions can disturb the formation of LDs, as it is regulated in MAM microdomains (40). Notably, as mentioned above, histological analysis of muscle tissues from *DEGS1* patients showed increases in the number and size of LDs in the cytosol. To confirm that LDs were larger in *DEGS1* patients, we stained patient fibroblasts with Oil Red O, a staining specific for neutral lipids and CE. We observed a significant increase in LD compartment size, as quantified by Feret’s diameter, and in the number of droplets per cell in patient fibroblasts (Figure 7, A–C).

LDs are composed of a core of neutral lipids and mainly consist of triacylglycerides (TAGs) and CE. TAGs are synthesized de novo from diacylglycerides (diacylglycerol [DAGs]) by diacylglycerol *O*-acyltransferase 1/2 (DGAT1/2), among others. When in excess, DAGs can also be stored in LDs (41). Notably, our targeted lipidomics data showed a significant increase in DAG levels in *DEGS1* patient fibroblasts. Additionally, the levels of its immediate biosynthetic precursor, phosphatidic acid (PA), were increased (Figure 7D). However, the levels of TAGs in *DEGS1* fibroblasts were not significantly increased compared with controls (Figure 7D).

To gain insight into the mechanism behind these alterations, we measured the expression of the genes encoding key enzymes involved in the regulation of DAGs and TAGs. We observed increased mRNA levels of *DGAT2* and diacylglycerol kinase alpha (*DGKA*), which phosphorylates DAGs into PA. There were no differences in the mRNA levels of *DGAT1* (Figure 7E). Of note, DGAT2, but not DGAT1, is localized at the MAM (42). These genes are controlled by the expression of master regulators of lipid homeostasis sterol regulatory element binding factors 1a, 1c, and 2 (*SREBF1a/1c/2*). The expression levels of these genes were also significantly increased in *DEGS1* patient fibroblasts (Figure 7F).

To validate the activation of SREBFs, we assessed the expression of its additional central target genes *HMGCS1*, *HMGCR*, *MVD*, and *SQLE*. All of these genes, which are involved in cholesterol synthesis, were upregulated in *DEGS1* fibroblasts (Figure 7G). SREBF2 expression is generally modulated by cholesterol-mediated negative feedback; in contrast, SREBF1 activation is not primarily controlled by cholesterol levels (43). In agreement with our SOAT1 activity assay results, we observed that the CE/FC ratio was decreased in *DEGS1* patient fibroblasts (Figure 7H), suggesting an impairment in the regulation of this enzyme. Therefore, increased FC levels do not inhibit the expression of SREBF2 in this context.

The expression and activation of SREBFs are also involved in the regulation of PL homeostasis. In *Drosophila melanogaster*, when PE levels were increased, the activation of SREBF1 was inhibited. In contrast, when PE levels were low, the SREBF pathway was stimulated (44). Moreover, in *Caenorhabditis elegans* and mammalian cells, phosphatidylcholine (PC) inhibited the activation of SREBF1. However, when PC synthesis was attenuated in *C*. *elegans,* human hepatoma cells, and mouse livers, the expression levels of SREBF and its target genes increased (45). Consequently, we evaluated the levels of the main mammalian PLs, PS, PE, and PC. In *DEGS1* patient fibroblasts, PS levels were increased, while the levels of PE were diminished and PC showed a decreasing trend (Figure 7I). These expected results are consistent with the PL synthesis and trafficking assay reflecting MAM activity shown in Figure 6, B and C.

Taken together, our data indicate that DEGS1 deficiency affects the regulation of TAG metabolism and TAG storage into LDs, as observed in muscle biopsies (Figure 1, A and D), as well as the regulation of PL homeostasis.

## Discussion

**Figure 8:**
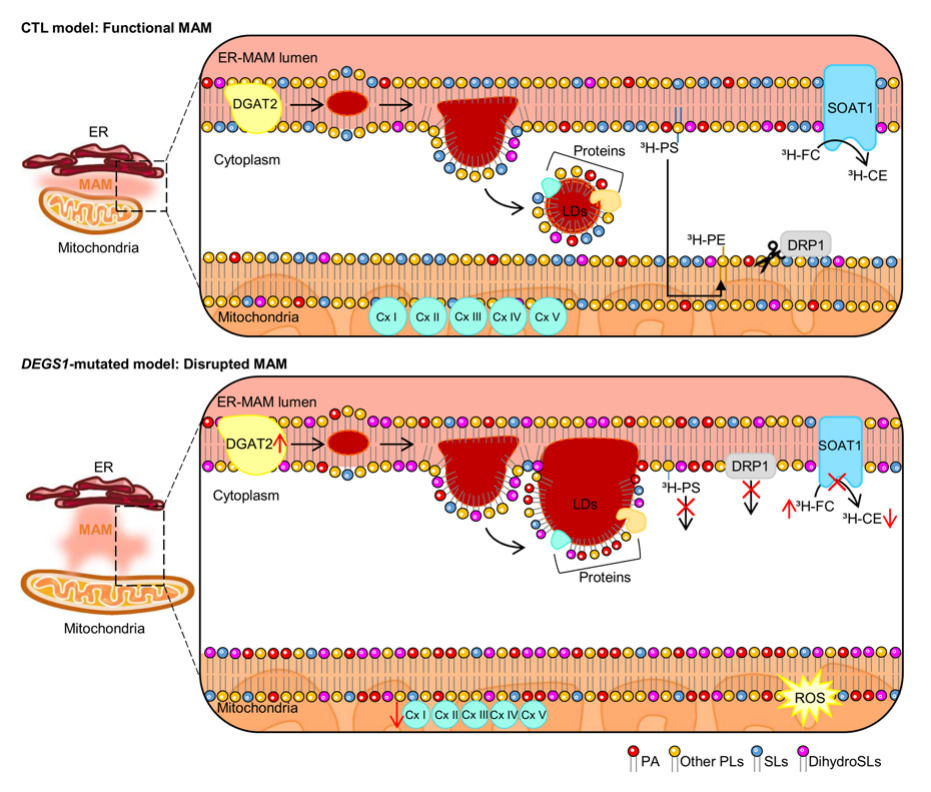
Schematic representation of the pivotal role of DEGS1 in the regulation of MAM and mitochondria function. As a consequence of DEGS1 loss, an imbalance in SL metabolism (DhCer, Cer, DhSM, SM, DhHexCer, HexCer) occurs (Figure 6, D–G). Since SLs are essential components of membranes, mitochondrial and ER-MAM appear physically disrupted and thus functionally impaired. MAMs cannot be properly formed, since the distance between mitochondria and the ER is greater than in controls (Figure 6, H and I). Moreover, 2 hallmarks of MAM function, PL transport/synthesis and CE synthesis, are decreased (Figure 6, B and C). The MAM components DRP1 and DGAT2 (42, 59), have their function affected leading to (a) decreased mitochondrial fission (Figure 3, A–F) and (b) increased size and numbers of LDs (Figure 7, A–C) due to DGAT2’s main role in LD formation (68). Mitochondria appear larger in size and hyperfused (Figure 1D, Figure 2, and Figure 3, A–D), with OXPHOS impairment (Figure 1C, Table 1, and Supplemental Table 2), decreased membrane potential (Figure 3G), and augmented production of superoxide anion levels (Figure 3H).

Biallelic loss-of-function mutations in *DEGS1* have been recently reported by our group and others as the genetic cause of HLD18 (9, 10, 11), but little is known about the mechanisms underlying the pathophysiology of this ultrarare disorder. Our results suggest that the disruption of the structure and function of the MAM is likely a key process underlying the metabolic and bioenergetic failure observed in *DEGS1* patient fibroblasts and tissues (Figure 8).

In this work, we show that, in contrast with what was shown in previous reports (10, 26, 27), DEGS1 is not homogeneously distributed in the ER, but is substantially enriched at MAM domains. This distribution is similar to that of other Cer-producing enzymes, such as sphingomyelinase and Cer synthases (Supplemental Figure 1) (18, 19, 21). Given that SLs are principal MAM constituents and essential components in lipid rafts, we posit that their imbalance may alter the MAM composition. In turn, disrupted MAMs may impair the correct synthesis of SLs in a vicious cycle, underscoring and expanding the critical role of MAMs in the regulation of SL metabolism and vice versa (46).

Our results also indicate that DEGS1 is necessary for both MAM integrity and its core functions in lipid metabolism, such as PL and CE biosynthesis (20, 37), which are severely affected in fibroblasts from *DEGS1* patients. These results expand the impact of DEGS1 deficiency beyond the SL pathway. Of note, the structure and activities of MAMs are intimately dependent on its lipidic composition. The MAM is a transient domain formed by a local elevation of cholesterol and SM in the ER, with the characteristics of a lipid raft (35, 47). This change in the lipid milieu of the ER induces the partitioning of lipid-binding ER proteins into discrete lipid domains and changes their conformation and functional regulation. Of direct relevance to this, defective DEGS1 causes an increase in the DhSM/SM ratio, as reported in this work and others (10, 11). Elevations in DhSM increase the rigidity of lipid raft microdomains (48). This is probably due to the enhanced affinity of DhSM for cholesterol in membranes, as shown by biophysical studies (49). MAM formation results in the activation of neutral sphingomyelinases, subsequent decreases in SM, and increases in Cer levels in MAMs and mitochondrial membranes (50, 51). Conversely, MAM-deficient cells present with decreased sphingomyelinase activity and lower SL turnover (52). Therefore, the functionality of MAMs and SL balance are intertwined, and DEGS1 is pivotal for their homeostasis.

Following on membrane composition, the myelin sheath is made of approximately 80% lipids and 20% proteins, with the principal constituent being GalCer (53). Of note, excess of GalCer due to the malfunction of galactosylceramidase causes demyelination in Krabbe disease (17). Because the MAM regulates the synthesis of Cer, which is the primary GalCer precursor, DEGS1 intrinsic dysfunction combined with disruption of the MAM may explain the defects in myelin formation and maintenance found in *DEGS1* patients.

Furthermore, we show that muscle tissue and fibroblasts from *DEGS1* patients exhibit a higher proportion of aberrantly elongated mitochondria, linked to an alteration in the regulation of mitochondrial dynamics, similar to that described in other primary mitochondria diseases, such as Leigh syndrome (54) or Charcot-Marie-Tooth type 2A (55). This, together with the impaired respiration in muscle biopsies and fibroblasts from patients, could be a consequence of altered mitochondrial morphology. Similar alterations occur in some mitochondriopathies in which cristae morphology defects are accompanied by OXPHOS dysfunction (28), as exemplified in optical atrophy 1 disease (OMIM #165500) (56). Defects in the regulation of the MAM in *DEGS1* patients could also help explain the mitochondrial hyperfused phenotype and bioenergetics impairment. An imbalance in the levels of Cer in ER-mitochondria connections has been suggested as inducing mitochondrial membrane damage and bioenergetic defects (57). Indeed, alterations in MAM protein recruitment and/or the lipidome of the mitochondrial outer and inner membranes can alter the control of the fusion and fission processes through DRP1 or MFN2 (30, 58, 59, 60). Mutations in *DRP1* cause optical atrophy 5 disease (OPA5) (OMIM #610708) and lethal encephalopathy (EMPF1) (OMIM #614388). Some patients with these *DRP1* mutations display decreased activity of complex IV (61) and complexes I, III, and IV (62) and defects in mitochondrial dynamics. In DEGS1 deficiency, however, MAM dyshomeostasis would induce a secondary impact on the mitochondrial shape and function of the OXPHOS complexes.

The MAM is involved in the regulation of PL synthesis and metabolism and in TAG and CE production (19, 42). Specifically, the MAM governs the synthesis of PA that is then transferred to mitochondria to produce cardiolipin (CL), a mitochondrial lipid that is essential for the maintenance of OXPHOS (63). Our data suggest that defects in MAM functionality may underlie the significant increases in the levels of PA that were observed in *DEGS1* patient fibroblasts. Notably, the mitochondrial fission protein DRP1 has a specific affinity for PA. This PL blocks DRP1 after its oligomerization onto mitochondria, leading to continuous assembly. Thus DRP1 is inhibited, which leads to a lower mitochondrial division/fission rate (63). Therefore, elevations in PA and a subsequent decrease in DRP1 activity could be the leading cause of the mitochondrial hyperfused phenotype that was observed in patient fibroblasts. Of note, PA itself can stimulate mitochondrial fusion by creating negative mitochondrial membrane curvatures (63). As a result of this hyperfused morphology, mitochondria may generate superoxides that in turn contribute to mitochondrial damage in a vicious cycle. In addition, PA is the immediate precursor of DAG synthesis in the glycerol-3-phosphate pathway. Therefore, impairments in the transfer of PA to mitochondria for CL production may promote a shift toward DAG synthesis, which could explain the higher DAG levels that were detected in *DEGS1* patient fibroblasts. Puzzlingly, despite these elevations in DAGs and in the expression of *DGAT2*, *DEGS1* patient fibroblasts showed normal levels of TAGs. Notably, DGAT2 is a MAM-resident enzyme whose expression is controlled by SREBFs, which are the master regulators of lipid metabolism (42). *SREBFs* were also increased in this context. One plausible explanation may be that SREBFs act as lipid biosensors in response to the decreased levels of PE and PC. Thus, SREBFs can cause an aberrant lipogenesis program and increase the formation and accumulation of LDs.

Finally, we showed the accumulation of supersized LDs in *DEGS1* patients, which could be a proxy for increased ROS formation, as proposed previously (64). Since MAM regulation has been suggested as a key factor in the formation of LDs (40), it is plausible that defects in MAM functionality could interfere with LD biogenesis in HLD18 pathogenesis. In the last decade, it has been reported that loss of LD homeostasis is disease causing in several monogenetic hereditary spastic paraplegias (65). However, whether LD malfunction is a contributing factor or rather plays a protective role in the context of broadly defined, complex neurodegenerative disorders is a matter of debate. Some authors posit an instrumental role of LDs in mitochondrially induced increased ROS and cellular demise (66), while others advocate for LDs as protective agents against potentially harmful lipids sequestered within, including Cer (67, 68).

This study highlights the importance of evaluating MAM and mitochondrial functionality in lipid disorders beyond primary mitochondriopathies. Taken together, our findings provide a conceptual basis for the underlying pathogenesis of HLD18 and may have implications for the management of this and other diseases caused by SL imbalance. The present study also exemplifies how rare monogenic diseases may help elucidate fundamental cellular metabolic functions. In this case, the knowledge gained could be applied to tackling more complex disorders, given the emerging role of DEGS1 in type 2 diabetes and cardiovascular disease (12, 13, 14, 15, 16).

## Methods

### Patients and genetic studies

Muscle biopsies were obtained from the quadriceps muscles of *DEGS1* patients and a 5-year-old child who served as a control. Skin-punch biopsies were performed to obtain primary human fibroblasts of *DEGS1* patients and controls. The fibroblasts were grown in DMEM (Thermo Fisher Scientific) containing 10% fetal bovine serum, 100 U/ml penicillin, and 100 μg/ml streptomycin and maintained at 37% in a humidified 95% air/5% CO_2_ incubator. Brain tissue (white matter of frontal lobes) from controls was obtained from the Brain and Tissue Bank for Developmental Disorders at the University of Maryland. More information about controls can be found in Supplemental Table 3.

### Mouse lines

Twelve-month-old male WT mice on a pure C57BL/6 background were used for all experiments. The mice were housed under a 12-hour light/12-hour dark cycle, with ad libitum access to food and water. The animals were sacrificed. The spinal cord was removed and stored at –80°C.

### Reagents

The reagents used are described in Supplemental Methods.

### Antibodies

The antibodies used for Western blots and immunofluorescence experiments are described in Supplemental Table 4. For DEGS1 colocalization with ERLIN2, these antibodies were conjugated with Alexa secondary antibodies. Briefly, anti-DEGS1 and anti-ERLIN2 antibodies were conjugated with Abberior STAR 635P, NHS ester, and Abberior STAR 580, NHS ester dyes, respectively. Briefly, the antibody-labeling reaction was performed by incubating a mixture containing the primary antibody, NaHCO_3_, and the appropriate pair of activator/reporter dyes diluted in DMSO for 40 minutes at room temperature (RT). Finally, purification of labeled antibodies was performed using NAP5 Columns (GE HealthCare).

### Histochemical muscle assays

All muscle biopsies were analyzed by an experienced neuropathologist. Muscular biopsies were processed according to standard procedures (69). Routine histology techniques performed are described in Supplemental Methods.

### TEM

Fresh muscle biopsies and growing fibroblasts were fixed in 2.5% glutaraldehyde (16210, Electron Microscopy Sciences) in 0.1M sodium cacodylate buffer (12310, Electron Microscopy Sciences), pH 7.1–7.4, for 2 hours at 4°C. Samples were washed 3 times in the same buffer and postfixed for another 2 hours at RT in 1% osmium tetroxide (19110, Electron Microscopy Sciences). After washing 3 times with the same buffer, dehydration was performed with an acetone series. Resin embedding in epoxy resin (18010, Ted Pella Inc.) was performed and encapsulated in the molds while orienting the sample. After polymerization for 48 hours at 60°C, we used a Leica EM Uc6 ultramicrotome to make ultrathin sections of the blocks of about 80 nm thick.

Slides were counterstained with 6% uranyl acetate (22400, Electron Microscopy Sciences) washed in tri-distilled water, incubated in 2% lead nitrate (17900, Electron Microscopy Sciences), and washed again in tri-distilled water. We observed the ultrathin sections with a Jeol JEM-1010 80 kv transmission electron microscope equipped with a CCD Orius camera (Gatan) with which we obtained the images.

#### Relative ER-mitochondria distance quantification.

We designed an ImageJ (NIH) macro to measure the shortest distance between 2 user-drawn regions of interest (ROIs) on an image, in this case, the mitochondria and the ER.

### Measurement of mitochondrial respiration chain function

#### Mitochondria enrichment.

Muscle mitochondria were isolated as previously described (70); see Supplemental Methods.

#### Polarographic study of substrate oxidation.

The oxidation of pyruvate was measured by the polarographic method as previously described (70, 71); see Supplemental Methods.

#### Spectrophotometric assays of respiratory chain enzyme activities.

Respiratory chain enzyme activities were quantified as previously described (70, 71); see Supplemental Methods.

### Immunofluorescence

Fibroblasts were seeded on coverslips prior to fixation, and MitoTracker Orange CMTMRos (M7510, Thermo Fisher Scientific) was used to stain mitochondria according to the manufacturer’s instructions. Briefly, 100 nM MitoTracker reagent in DMEM was added to the culture. After 15 minutes of incubation at 37°C, the cells were washed 3 times with PBS. Fibroblasts were fixed for 30 minutes with 2 ml per well of 10% formalin at RT. To permeabilize and block the cells, coverslips were incubated for 20 minutes at RT in blocking buffer (1% BSA, 0.2% powdered milk, 2% NCS, 0.1M glycine, 0.1% Triton X-100). Fibroblasts were immunostained with the indicated primary antibodies overnight at 4°C. Following incubation with secondary antibodies for 1 hour at RT, the slides were mounted using Mowiol. The slides were analyzed with an SP5 confocal microscope (Leica TCS). Confocal images were acquired using a Leica TCS SL laser scanning confocal spectral microscope (Leica Microsystems), and images were analyzed with ImageJ. Mitochondria morphology (area and sphericity) and Manders’ correlation coefficient were quantified using Imaris software, version x64 9.7.2. The 3D image reconstruction of patient fibroblasts was also performed using Imaris software.

#### Live-cell imaging.

Fibroblasts were seeded in μ-Slide 8-Well High (80806, Ibidi) chambers. Growing fibroblasts were stained with MitoTracker as previously described. We optimized the conditions to avoid photobleaching, decreased the concentration of MitoTracker to 50 nM, and decreased laser intensity to the minimum. 3D images of fibroblasts were acquired using a Leica TCS SP8 STED 3× Laser Scanning Confocal Microscope (LSCM) with an HC PL APO CS2 ×100 oil objective (pixel size = 113 nm). Fluorescence excitation was achieved with a pulsed white-light laser source, and fluorescence emission was collected using a Leica HyD SMD detector. Images were obtained every 16 minutes for 4 hours and analyzed with Imaris software by means of the disconnected number of counts for automatic quantification of mitochondria units over time.

### Inner ΔΨm quantification

Growing fibroblasts were washed with PBS and incubated with 50 nm tetramethylrhodamine, ethyl ester (TMRE) (Molecular Probes) in prewarmed PBS for 30 minutes at 37°C. Cells were trypsinized, centrifuged at 1,000*g* for 5 minutes, and resuspended in prewarmed PBS. All samples were captured by a Gallios Analyzer (Beckman Coulter), which recorded 20,000 cells for each genotype tested. FCCP (200 μM for 10 minutes) was used as a positive control. Histograms showing the inner Ψm levels were obtained after gating live cells. The data were analyzed with Kaluza Analysis software, version 2.1. (Beckman Coulter).

### Evaluation of ROS production

Intracellular and mitochondrial superoxide anion levels were estimated using DHE and MitoSOX Red probes (Thermo Fisher, D11347 and M36008), respectively. Briefly, after incubating with 5 μM DHE or MitoSOX for 10 minutes, fibroblasts were washed twice with PBS and scraped into water. The homogenate was transferred into a 96-well plate for fluorescence detection with a spectrofluorimeter. The fluorescence of DHE- and MitoSOX-stained cells was measured with a spectrofluorometer (FLUOstar Omega Microplate Reader, BMG Labtech). The excitation wavelength was 530 nm, and the emission wavelength was 590 nm for DHE and MitoSOX. Fluorescence values were corrected with protein content, which was measured using a Pierce BCA Protein Assay Protocol (Thermo Fisher). Antimycin A (MilliporeSigma, A8674) was used as a positive control.

### MAM collection

Purification of the ER, MAM, and mitochondria was performed and results were analyzed as previously described (72). On average, 370.47 mg of frozen human brain white matter was used. In mice, a pool of 6 spinal cords per *n* had to be made, so we used the spinal cords of 30 mice to obtain *n* = 5.

### Western blot

Human fibroblasts were homogenized in RIPA buffer (150 mM NaCl, 1% Nonidet P40, 0.5% sodium deoxycholate, 0.1% SDS, 50 mM Tris, pH 8.0), sonicated for 1 minute at 4°C, centrifuged for 10 minutes at 1,000*g*, mixed with 4× NuPAGE LDS Sample Buffer (Invitrogen), and heated for 10 minutes at 70°C. Protein amounts were quantified using a BCA Protein Assay Kit (Thermo Fisher Scientific). Protein samples (25 μg) were subjected to polyacrylamide gel electrophoresis for 1 hour at 120 V in NuPAGE MOPS SDS Running Buffer (Invitrogen) supplemented with 5 mM sodium bisulfite (Sigma-Aldrich, 243973). Proteins were transferred to nitrocellulose membranes using an iBlot 2 Gel Transfer Device (Invitrogen). After blocking with 5% BSA (Sigma-Aldrich) in 0.05% TBS-Tween (TBS-T) for 1 hour at RT, membranes were incubated with primary antibodies overnight at 4°C. Following incubation with secondary antibodies for 1 hour at RT, proteins were detected with a Chemidoc Touch Imaging System (Bio-Rad). Bands were quantified with ImageLab (Bio-Rad).

### Proteomics analysis

Sample preparation, mass spectrometry (MS) analysis, bioinformatics, and data evaluation of MAM samples were performed in collaboration with IDIBELL’s Proteomics Platform. Briefly, 10 μg of MAM lysates in lysis buffer were digested with Lys-C and Trypsin. Prior to digestion, samples were reduced and alkylated with DTT and CAA; then the samples were diluted with Tris 0.1M to reach urea 2 mol/L. Lys-C was added at 1:25 (w/w) (enzyme-to-protein ratio), and protein digestion was carried out at 30°C overnight. Then the samples were diluted again with Tris 0.1M to reach urea 0.8 mol/L. Trypsin was added at 1:25 (w/w) (enzyme-to-protein ratio), and protein digestion was carried out at 30°C over 8 hours. Enzymatic reaction was stopped with FA (10% [v/v] final concentration). Digested samples were desalted using C18.

Liquid chromatography–MS (LC-MS) analysis was carried out using an HPLC system (EASY-nanoLC 1000, Thermo Scientific), with a C18 column of 50 cm (EASY-Spray; 75 μm ID, PepMap RSLC C18, 2 μm particles, 45°C) and a 90-minute gradient connected to a Orbitrap Fusion Lumos Mass Spectrometer (Thermo Scientific). The raw data were analyzed using the Proteome Discoverer software suite (version 2.0, Thermo Fisher Scientific), and the Mascot search engine (version 2.5, Matrix Science) was used for peptide identification and quantification. Samples were searched against a SwissProt database containing entries corresponding to human (version of January 2018), a list of common contaminants, and all the corresponding decoy entries. Trypsin was chosen as an enzyme, and a maximum of 3 miscleavages were allowed. Carbamidomethylation (C) was set as a fixed modification, whereas oxidation (M) and acetylation (N-terminal) were used as variable modifications. Searches were performed using a peptide tolerance of 7 ppm and a product ion tolerance of 0.5 Da. Resulting data files were filtered for FDR of less than 1%.

### PhotoClick cholesterol assay

To further study the MAM resident proteins, a method developed by Hulce et al. (36) was used. Briefly, MEFs were incubated in serum-free medium for 2 hours to remove all exogenous lipids. After that, 5 μM photoClick cholesterol (Hex-50-ynyl 3b-hydroxy-6-diazirinyl-5a-cholan-24-oate), previously complexed with an aqueous saturated solution of MβCD (38 mM), was added to the cells and incubated for 4 hours. Upon washing with DPBS, photoClick cholesterol was crosslinked under 365 nm-UV (0.75 J/cm^2^, UVC 500 Ultraviolet Crosslinker; Amersham Biosciences), washed again, collected, and used for subcellular fractionation as described above. Protein (500 μg) (adjusted to a final 150 μl volume with PBS supplemented with protease inhibitors) of the MAM fraction was briefly sonicated and subjected to click chemistry by the addition of 500 μM biotin-azide, 100 μM Tris([1-benzyl-1H-1,2,3-triazol-4-yl]methyl)amine (TBTA), 1 mM CuSO_4_, and 1 mM Tris(2-carboxyethyl)phosphine (TCEP). It was incubated for 15 minutes at RT in the dark. Then samples were diluted in 50 mM Tris pH 7.4 with protease inhibitors (removing an aliquot as input) and incubated with streptavidin beads overnight under rotation at 4°C. After several washes with 50 mM Tris pH 7.4, the beads were collected by centrifugation at 2,000*g* for 1 minute, boiled with NuPAGETM LDS Sample Buffer (1×) at 95°C for 5 minutes, and used for immunodetection.

### PL synthesis and trafficking assay

Fibroblasts were incubated for 2 hours with serum-free medium to ensure the removal of exogenous lipids. The medium was then replaced with MEM containing 2.5 μCi/ml ^3^H-serine for the indicated periods of time. Fibroblasts were washed and collected in DPBS. They were pelleted at 2,500*g* for 5 minutes at 4°C and resuspended in 0.5 ml water, removing a small aliquot for protein quantification. Lipid extraction was performed by the Bligh and Dyer method. Briefly, 3 volumes of chloroform/methanol 2:1 were added to the samples and vortexed. After centrifugation at 8,000*g* for 5 minutes, the organic phase was washed twice with 2 volumes of methanol/water 1:1. Then, the organic phase was evaporated under nitrogen to dryness. Dried lipids were resuspended in 60 μl of chloroform/methanol 2:1 (v/v) and applied to a TLC plate. PLs were separated using 2 solvents composed of petroleum ether/diethyl ether/acetic acid 84:15:1 (v/v/v) and chloroform/methanol/acetic acid/water 60:50:1:4 (v/v/v/v). Development was performed by exposing the plate to iodine vapor. The spots corresponding to the relevant PLs (identified using comigrating standards) were scraped and counted in a scintillation counter (Packard Tri-Carb 2900TR).

### SOAT1 activity assay

To measure SOAT1 activity in vivo, whole fibroblasts were incubated in serum-free medium for 2 hours to remove all exogenous lipids. After that, 2 μCi/ml ^3^H-cholesterol was added to FBS-free DMEM containing 2% FAF-BSA and allowed to equilibrate for at least 30 minutes at 37°C. Then the radiolabeled medium was added to the cells for the indicated periods of time. Fibroblasts were then washed and collected in DPBS, removing a small aliquot for protein quantification. Lipids were extracted as described above, and samples were analyzed by TLC along with an unlabeled CE standard. A mixture of chloroform/methanol/acetic acid 190:9:1 (v/v/v) was used as a solvent. Iodine stains corresponding to CE bands were scraped and counted.

### Lipidomics profiling

Lipidomics experiments were performed at the Lipidomics Facility of Columbia University and analyzed by LC-MS. Lipids were separated via chloroform-methanol extraction and analyzed with a 6490 Triple Quadrupole LC-MS system (Agilent Technologies) according to the manufacturer’s instructions. Glycerophospholipids were separated with normal-phase HPLC using an Agilent Zorbax Rx-Sil column (inner diameter, 2.1 × 100 mm).

### Oil Red O staining in fibroblasts

A concentrated solution was prepared by dissolving 100 mg Oil Red O (Sigma-Aldrich, O0625) in 12 ml of triethyl phosphate of 99.8% or more (Sigma-Aldrich, 538728) and 8 ml of distilled H_2_O. The resulting solution was stirred overnight at RT. After filtering with a 0.2 μm filter, 15 ml of the concentrated solution was diluted with 10 ml of distilled H_2_O to obtain the working solution. The medium was removed from the wells with a glass pipette, and the fibroblasts were rinsed once with PBS. Fibroblasts were fixed for 30 minutes with 2 ml per well of 10% formalin at RT. After fixing, the wells were rinsed with PBS 3 times for 30 seconds each. Prior to use, the coverslips were rinsed once with distilled H_2_O. Oil Red O working solution was applied (2 ml per well) for 1.5 hours at RT. The staining solution was removed, and the cells were washed twice with distilled H_2_O for 1 minute. Hematoxylin (2 ml per well) was added for 10 minutes to counterstain the nuclei. This was followed by one more rinse with distilled H_2_O for 1 minute. The coverslips were allowed to dry completely at RT and then mounted with 12 ml Mowiol. The Feret’s diameter function of ImageJ software was used to determine the LD diameters. This parameter measures the longest distance between any 2 points along the selection boundary and is also known as the maximum caliper. ImageJ software was also used to count the number of LDs per cell. Between 30 and 50 fibroblasts per clone were quantified.

### Quantitative real-time PCR

Total RNA was extracted using the RNeasy Mini Kit according to the manufacturer’s instructions. 1 μg of RNA was transcribed into complementary DNA (cDNA) using SuperScript IV reverse transcriptase in a final volume of 25 μl. TaqMan real-time PCR and SYBR Green real-time PCR were performed in the LightCycler 480 Real-Time PCR System (Roche Diagnostics). Primers used are described in Supplemental Table 5. The expression of the genes of interest was normalized to that of the human reference gene *RPLP0*. Each sample was run in triplicate, and the mean value was used to calculate mRNA expression using the comparative (2^−ΔCt^) method, according to the manufacturer’s instructions.

### Statistics

Statistical significance was assessed using Student’s *t* test or Wilcoxon’s test when 2 groups (CTL versus DEGS1^mut^) were compared using parametric and nonparametric tests, respectively. *P* < 0.05 was considered statistically significant. Manders’ correlation coefficient was performed using ImageJ software, and 2-tailed Student’s *t* test and Wilcoxon’s tests were performed using R software.

### Study approval

All procedures involving human and animal samples were approved by the Animal Experiments Committee of IDIBELL (PR076/14). All animal experiments were conducted following protocols approved by the Animal Experiments Committee at the Generalitat de Catalunya. Written, informed consent was obtained for all affected children from parents or legal guardians.

Author contributions

LPS designed, performed, and analyzed the results of most of the experiments, assembled the figures, and wrote the manuscript. NL assisted in the immunofluorescence assays. MPO and EAG developed the MAM functional assays and lipidomics assays. LG performed the ROS experiments. CDLT performed proteomics assays. In-depth clinical and pathological characterization and recruitment of patients were carried out by CJ, NJP, AGC, AG, BH, AF, IF, MR, CC, and SF. MM and PLA assisted in confocal studies. AP designed and supervised the overall research and wrote the manuscript. All authors critically reviewed the report and approved the final version.

## Supplementary Material

Supplemental data

Supplemental video 1

Supplemental video 2

Supplemental video 3

Supplemental video 4

## Figures and Tables

**Table 1:**
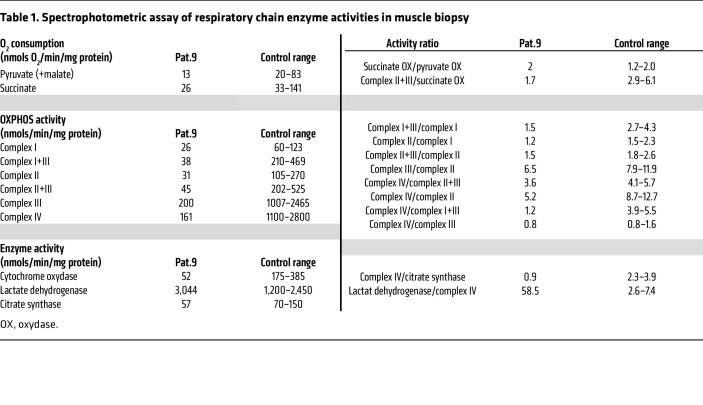
Spectrophotometric assay of respiratory chain enzyme activities in muscle biopsy

**Table 2:**
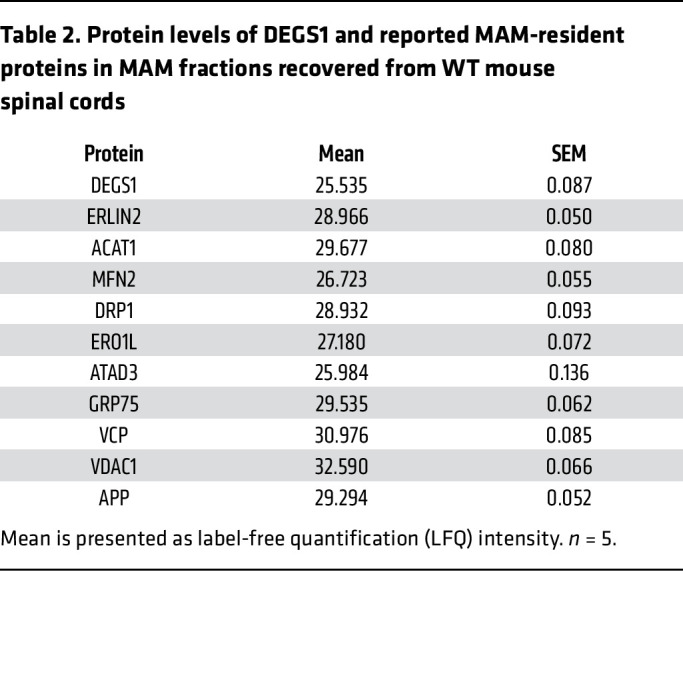
Protein levels of DEGS1 and reported MAM-resident proteins in MAM fractions recovered from WT mouse spinal cords

**Table 3:**
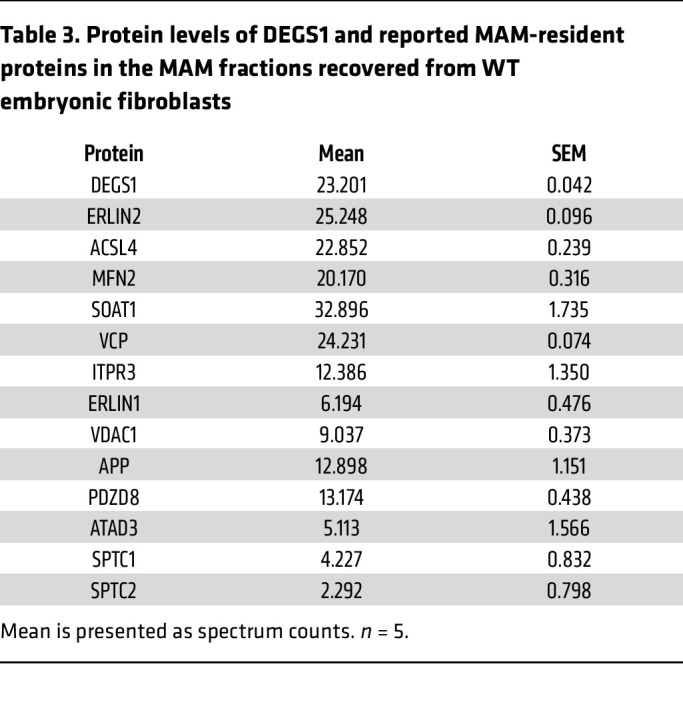
Protein levels of DEGS1 and reported MAM-resident proteins in the MAM fractions recovered from WT embryonic fibroblasts

## Version 1 03/23/2023

In-Press Preview

## Version 2 05/15/2023

Electronic publication

## Version 3 05/24/2023

Renamed supplemental videos
