# Supplementary material for: Sphingolipid desaturase DEGS1 is essential for mitochondria-associated membrane integrity
Source: J Clin Invest. 2023 May 15;133(10):e162957. doi: 10.1172/JCI162957 (PMC10178845; doi:10.1172/JCI162957)
Supplement: Supplemental data [file jci-133-162957-s012.pdf]

SUPPLEMENTAL MATERIAL

MATERIALS & METHODS

Reagents

The following reagents were purchased from Sigma-Aldrich: D-mannitol (M4125), ethylene glycol-bis(2-aminoethyl ether)-N, N, N', N'-tetraacetic acid (EGTA) (E4378), N-(2-hydroxyethyl)piperazine-N'-(2-ethanesulfonic acid) (HEPES) (H3375), Percoll® (P4937) and Bis-Tris (B7535). Protease inhibitor cocktail tablets (11836153001) were purchased from Roche Diagnostics. The SuperScript IV First-Strand cDNA Synthesis Reaction kit was purchased from Life Technologies. The RNeasy Mini Kit was purchased from Qiagen, and the SuperScript IV reverse transcription reagent was purchased from Invitrogen™.

Histochemical muscle assays

Sudan Black staining

10 µm thickness sections were cut and incubated for 7 minutes in saturated Sudan Black (Sigma Aldrich) solution. After incubation, sections were rinsed with running water and distilled water. Preparations were mounted with aqueous mounting medium.

Oil Red O staining

12 µm thickness sections were cut and fixed for 1 hour with paraformaldehyde at 4ºC. After fixation, sections were rinsed with distilled water and incubated for 1 minute with isopropanol 60%. After this short incubation, sections were incubated with Oil Red O solution for 30 minutes. Oil Red O stock solution was prepared with 2.5 gr of Oil Red O

(Merk Millipore) in 500 mL of Isopropanol (Merk Millipore). For incubation, three parts of Oil Red O stock were diluted with two of distillated water. After incubation, sections were rinsed with running water and distillated water. Preparations were mounted with aqueous mounting medium.

Hematoxylin-eosin staining

10 µm thickness sections were cut and incubated for 8 minutes with hematoxylin, rinsed abundantly with running water and then briefly incubated with eosin for 30 seconds followed of dehydration and DPX mounting.

Modified Gömöri Trichrome staining

10 µm thickness sections were cut and incubated for 8 minutes with hematoxylin, rinsed abundantly with running tap water and then incubated for 45 minutes in Red Mallory solution. After that, slides were rinsed abundantly in distillated water and incubated in phosphotungstic acid for 3 minutes and then, without washing, directly incubated in fast green for 40 minutes. Finally, slides were abundantly rinsed in running tap water followed of dehydration and DPX mounting.

Cytochrome C Oxidase (COX) histochemistry

10 µm thickness sections were cut and incubated for 3 hours at room temperature with COX solution. Stock COX solution was prepared with 8ml sodic phosphate 0.2M (Sigma Aldrich), 2 ml of potassium phosphate 0.2 M (Merk Millipore), 11 mg of diaminobencidine (Sigma Aldrich) 100 mg of catalase (Sigma Aldrich), 750 mg of sucrose (Sigma Aldrich) and 10 mg cytochrome C (Sigma Aldrich). Stock solution is aliquoted in 500µl eppendorfs and stored at -80º C. After incubation, sections were fixed

for 5 minutes with calcic formaldehyde and rinsed with distillated water followed of dehydration and DPX mounting.

Succinate Dehydrogenase (SDH) histochemistry

10 µm thickness sections were cut and incubated for 2.5 hours at 37ºC with SDH solution. SDH stock solution was prepared with 4 ml sodic phosphate 0.2M (Sigma Aldrich), 1 ml of potassium phosphate 0.2 M (Merk Millipore), 5 ml of sodic succinate (2.7 gr of succinate in 50 ml of distillated water) (Sigma Aldrich) 10 mg of Nitro Blue tetrazolium (Sigma Aldrich). Stock solution is aliquoted in 500µl eppendorfs and stored at -80º C. After incubation, sections were fixed for 5 minutes with calcic formaldehyde, rinsed with distillated water and mounted in aqueous mounting medium.

Measurement of mitochondrial respiration chain function

Mitochondria enrichment

Briefly, 100 mg of muscle tissue was minced with scissors in 1.5 ml of medium A (20 mM Tris-HCl pH 7.2, 0.25 M sucrose, 40 mM KCl, 2 mM EGTA, and 1 mg/ml BSA). After homogenization by 7 strokes in a glass-Teflon potter, the homogenate was filtered through a 90 µm nylon net. Then, 100 µl of filtrate and all residual material on the filter were set aside to later perform respiratory chain enzyme activities. The filtrate was centrifuged for 8 min at 2,000 x g. The supernatant was set aside, and the pellet was resuspended in 1 ml of medium A. The resuspended pellet was homogenized by 7 strokes in a glass-Teflon potter. It was then centrifuged for 8 min at 2,000 x g and pooled with the first supernatant. The supernatant was centrifuged for 8 min at 10,000 x g. The pellet was resuspended in 1 ml of medium A and centrifuged again for 8 min at 10,000 x g.

After centrifugation, the pellet of washed mitochondria was resuspended in 30 µl of medium A to measure mitochondrial enzymatic activities.

Polarographic study of substrate oxidation

Briefly, 0.5 µg of washed mitochondria was added to 250 µl of medium B (0.3 M mannitol, 10 mM KCl, 5 mM $MgCl_2$, 10 mM KH_2PO_4, and 1 mg/ml BSA) in the presence of 10 mM pyruvate, 500 µM malate, 0.3 mM ADP and 10 mM succinate at 37 ºC, pH 7.4. The oxidation of succinate was measured by the polarographic method. Briefly, 0.5 µg of washed mitochondria was added to 250 µl of medium B (0.3 M mannitol, 10 mM KCl, 5 mM $MgCl_2$, 10 mM KH_2PO_4, and 1 mg/ml BSA) in the presence of 0.2 mM ATP and 10 mM succinate at 37 ºC, pH 7.4.

Spectrophotometric assays of respiratory chain enzyme activities

Cytochrome c oxidase (complex IV) was quantified at 550 nm with 0.05 µg of washed mitochondria in 1 ml of medium E (0.3 M sucrose, 10 mM KH_2PO_4, 1 mg/ml BSA) in the presence of 10 µM reduced cytochrome c (cyt c) at 37 ºC, pH 6.5. The activity of complexes II + III was quantified at 550 nm with 0.05 µg of washed mitochondria in 1 ml of medium D (10 mM KH_2PO_4, 1 mg/ml BSA, 2 mM EDTA) in the presence of 40 µM oxidized cyt c, 3 µM rotenone, 10 mM succinate, 0.2 mM ATP and 0.3 mM KCN at 37 ºC, pH 7.8. Then, complex II was inhibited by 10 mM malonate, and complex III was quantified at 550 nm in the presence of 50 µM ubiquinol. Then, 0.05 µg of washed mitochondria was incubated for 3 min in 800 µl of distilled water. The activity of complexes I + III was quantified at 550 nm in the presence of 200 µl medium F (50 mM Tris-HCl, 5 mg/ml BSA), 40 µM oxidized cyt c, and 0.8 mM NADH at 37 ºC, pH 8.0. The activity of complex II was quantified at 600 nm with 0.05 µg of washed mitochondria

in 1 ml of medium D (10 mM KH_2PO_4, 1 mg/ml BSA, 2 mM EDTA) in the presence of 40 µM oxidized cyt c, 3 µM rotenone, 10 mM succinate, 0.2 mM ATP, 0.3 mM KCN, 80 µM dichlorophenol indophenol, 1 µM antimycin A and 50 µM decyl ubiquinone at 37 ºC, pH 7.8. Then, 0.3 µg of washed mitochondria was incubated for 3 min in 800 µl of distilled water, and the activity of complex I was quantified at 340 nm in the presence of 200 µl medium F (50 mM Tris-HCl, 5 mg/ml BSA), 0.8 mM NADH, 50 µM decyl ubiquinone, and 3 mM KCN at 37 ºC, pH 8.0.

VIDEOS

Supplemental Video 1. Live-cell imaging_CTLchild_1. Mitochondrial morphology by MitoTracker staining in 1-hour live-cell imaging in control fibroblasts; disconnected mitochondria are each shown in different colours.

Supplemental Video 2. Live-cell imaging_CTLchild_2. Mitochondrial morphology by MitoTracker staining in 1-hour live-cell imaging in control fibroblasts; disconnected mitochondria are each shown in different colours.

Supplemental Video 3. Live-cell imaging_DEGS1mut_1. Mitochondrial morphology by MitoTracker staining in 1-hour live-cell imaging in *DEGS1* patient fibroblasts; disconnected mitochondria are each shown in different colours.

Supplemental Video 4. Live-cell imaging_DEGS1mut_2. Mitochondrial morphology by MitoTracker staining in 1-hour live-cell imaging in *DEGS1* patient fibroblasts; disconnected mitochondria are each shown in different colours.

FIGURES

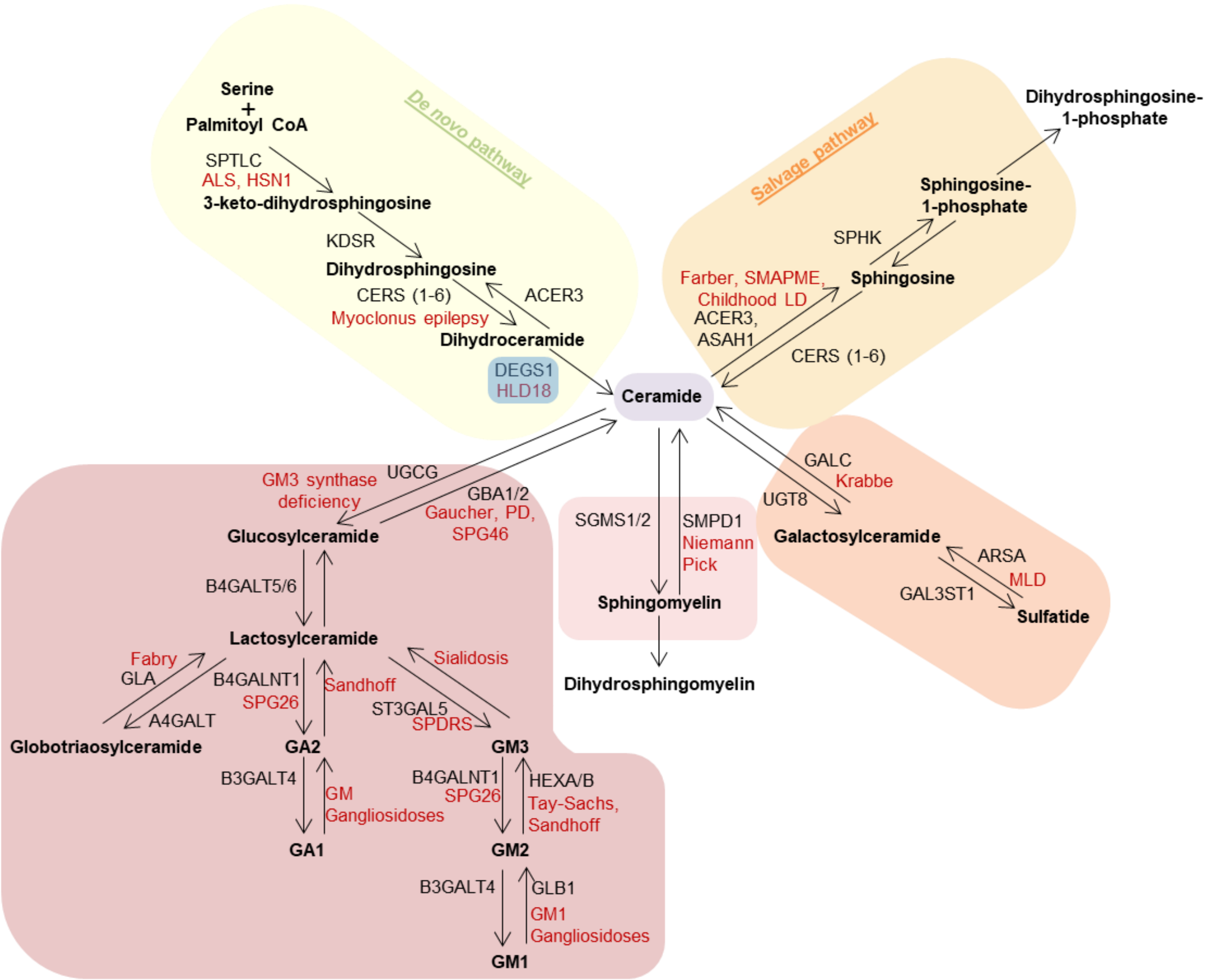

Supplemental Figure 1. Scheme depicting enzyme defects of the sphingolipid pathway causing neurological disorders.

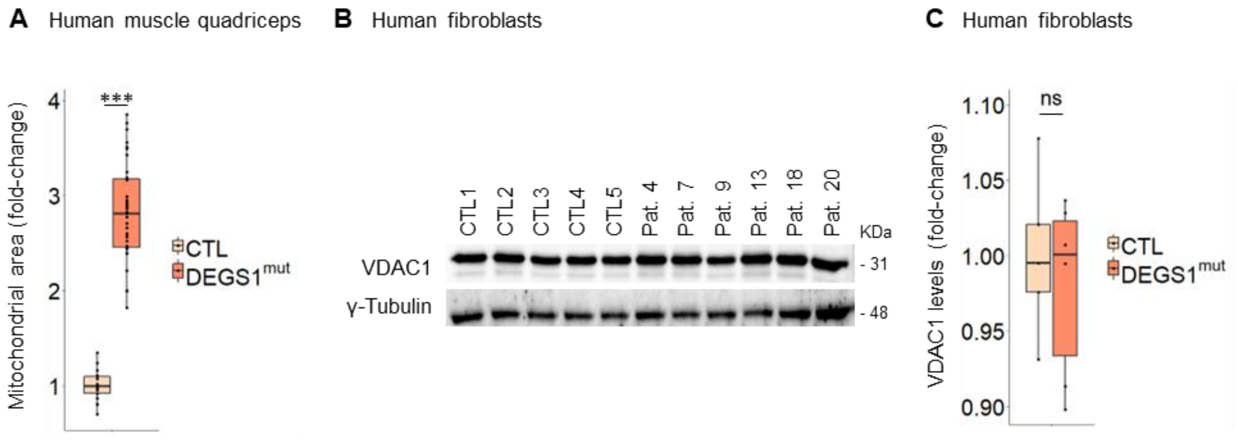

Supplemental Figure 2. (A) Mitochondrial area quantification in quadriceps muscle biopsy from Pat. 9 and control child. **(B)** Western blot analysis of mitochondrial proteins and its **(C)** quantification. Data are presented as box-and-whisker plots (median, interquartile interval, minimum, maximum). ****P<0.001* (2-tailed Student's *t test*).

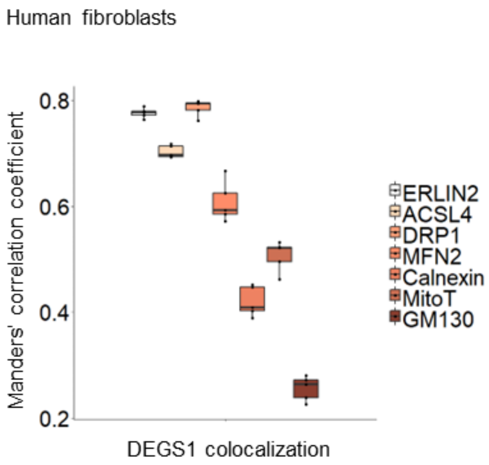

Supplemental Figure 3. Manders' correlation coefficient was used to quantify the colocalization degree between DEGS1 and the different markers. The experiment was done in triplicates. Data are presented as box-and-whisker plots (median, interquartile interval, minimum, maximum).

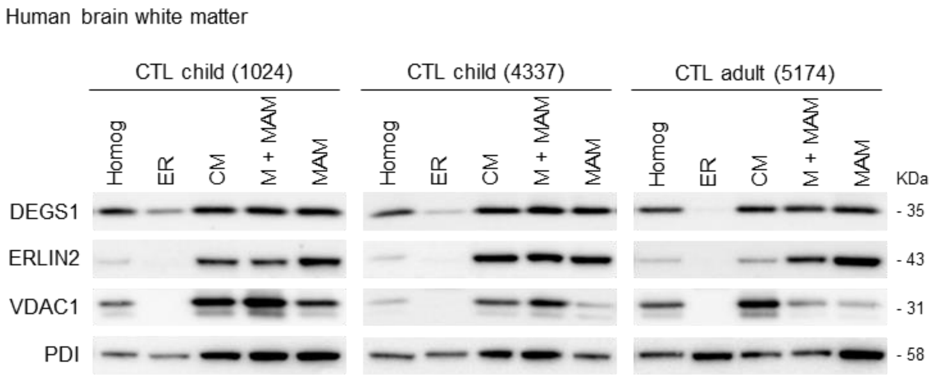

Supplemental Figure 4. Western blot analysis of all recovered fractions during the MAM enrichment collection from human brain white matter.

TABLES

Supplemental Table 1. Clinical description of patients 4, 7, 9, 13, 18 and 20.

**Patient number**	**Pat. 4**	**Pat. 7**	**Pat. 9**	**Pat. 13**	**Pat. 18**	**Pat. 20**
Mutations	c.341_342delTT/764A>G p.(Leu114Profs*11)/(Asn255Ser)	c.764A>G p.(Asn255Ser)	c.337A>G p.(Asn113Asp)	c.320G>A p.(Trp107*)	c.320G>A p.(Trp107*)	c.518G>C/601dupT p.(Arg173Pro)/(Tyr201LeufsTer7)
Age of onset (months)	6	24	1	1	1	1
Sex	Female	Male	Female	Male	Female	Female
Neurological signs	hypomyelination, limb dystonia, spasticity	hypomyelination, spasticity, dysmetria	hypomyelination, limb dystonia, spasticity	hypomyelination, limb dystonia, spasticity	hypomyelination, spasticity	hypomyelination, axial hypotonia, spastic tetraparesis, orolingual dystonia, acquired microcephaly: 46,5cm (-4 SD, at 9y)
Seizures (onset age)	clonic tonic severe (22 m) status epilepticus; stop ketogenic diet (5y)	none	clonic tonic (8m) with status epilepticus	tonic (2,5y)	febrile (4y)	none, EEG (8y): multifocal seizure epileptic discharges
Language acquisition (age)	few words (1y)	sentences (5y) simple reading	none	none	none	none
Motor development score	1	4	1	0	0	0
Eye abnormalities	nystagmus and abnormal saccades; mild optic atrophy	none	nystagmus (1m), 1 episode of tonic-upgaze like (8y), ERG normal	nystagmus (1m), ERG normal	nystagmus (1m)	oculogyric crisis (5m), bilateral mydriasis
Gastrostomia	yes (2y; gain)	no	no	feeding difficulties	no	yes (8y)
Other signs	failure to thrive (-4 SD W; -4 SD H), premature pubarche kyphosis and scoliosis	none	failure to thrive (-3.5 SD W; -4 SD H), gingival hypertrophy, scoliosis, and hip dislocation > 4,5 years of age	none	failure to thrive (-3.5 SD W; -6 SD H)	failure to thrive (-30 SD W; -6 SD H, at 10y), dysphagia, hypothyroidism, bilateral hip dysplasia, genu valgum scoliosis, frequent respiratory infections

Supplemental Table 2. Spectrophotometric assay of respiratory chain enzyme activities in fibroblasts biopsies.

**O2 consumption (nmols O2/min/mg protein)**	**Pat.9**	**Control range**
Pyruvate (+malate)	3.7	3.3 - 6.8
Succinate	13	6.5 - 14.3
Glycerol-3-phosphate	6.5	3.5 - 6.7
Decylubiquinol	19	8.5 - 23.2

**OXPHOS activity (nmoles/min/mg protein)**	**Pat.9**	**Control range**
Complex II	17	10.8 - 17
Complex II+III	**20**	21 - 42
Complex III	**70**	98 - 180
Complex IV	91	72 - 143
G3P dehydrogenase	13	9.2 - 17.7
G3P dehydrogenase + Complex III	11	8.3 - 28

**Enzyme activity (nmoles/min/mg protein)**	**Pat.9**	**Control range**
Lactate dehydrogenase	**5736**	1960 - 4360
Citrate synthase	**79**	32 - 72

**Activity ratio**	**Pat.9**	**Control range**
Succinate OX / Pyruvate OX	**3.5**	1.3 - 2.5
Succinate OX / Gly3O OX	2	1.4 - 3.0
Duroquinol OX / Succinate OX	1.5	1.1 - 1.9
Complex IV / Complex II	5.4	4.2 - 7.8
Complex IV / Complex II+III	4.5	2.2 - 4.6
Complex IV / Complex III	1.3	0.6 - 1.4
Complex II+III / Succinate OX	**1.5**	2.3 - 3.9
Complex III / Duroquinol OX	**3.7**	5.7 - 10.5
Complex II+III / G3P dehydr + Complex III	1.8	1.0 - 2.6
Complex II / G3P dehydr	1.3	0.8 - 1.6
Complex IV/ Citrate synthase	1.2	1.2 - 2.8
Lactat dehydrogenase / Complex IV	**63**	13 - 53

**OXPHOS activity (nmoles/min/mg protein)**	**Pat.20**	**Control range**
Complex I+III	38	15.0 - 42.0
Complex II	25,8	22.0 - 35.0
Complex II+III	**30.6**	11.0 - 20.0
Complex III	36,1	25.0 - 48.0
Complex IV	**64.5**	33.0 - 57.0

**Activity ratio**	**Pat.20**	**Control range**
Complex I+III/ citrate synthase	0.51	0.24 - 0.85
Complex II/ citrate synthase	**0.34**	0.53 - 0.75
Complex II+III/ citrate synthase	0.41	0.25 - 0.42
Complex III/ citrate synthase	**0.48**	0.49 - 1.06
Complex IV/ citrate synthase	0.86	0.84 - 1.15

**Enzyme activity (nmoles/min/mg protein)**	**Pat.20**	**Control range**
Citrate synthase	**74.9**	41.0 - 62.0

Supplemental Table 3. Description of control samples.

ID	Gender	Age (years)	Race	Tissue	Observations
CTL1	Male	16	Caucasian	Fibroblasts	Passage 13-16
CTL2	Male	17	Caucasian	Fibroblasts	Passage 13-16
CTL3	Male	18	Caucasian	Fibroblasts	Passage 14-17
CTL4	Male	26	Caucasian	Fibroblasts	Passage 13-16
CTL5	Male	22	Caucasian	Fibroblasts	Passage 13-16
1024	Male	14	Caucasian	Brain	Postmortem interval (hours): 16
4337	Male	8	Caucasian	Brain	Postmortem interval (hours): 16
5174	Male	61	Caucasian	Brain	Postmortem interval (hours): 21
5114	Male	37	Caucasian	Brain	Postmortem interval (hours): 9
CTL	Male	5	Caucasian	Musle	Quadriceps biopsy

Supplemental Table 4. Antibody specifications.

Antibody	Dilution	Source	Identifier
Calnexin	IF: 1/100	Novus Biologicals	NB300-518
Complex I	IHC: 1/100	Life Technology Invitrogen®	ab459210
DEGS1	WB: 1/1000, IF: 1/50	Abcam®	ab167169
DRP1	WB: 1/500, IF: 1/50	Cell Signaling Technology®	14647
pDRP1^{S616}	WB: 1/500, IF: 1/50	Cell Signaling Technology®	3455
ACSL4 Recombinant Alexa Fluor® 488	IF: 1/50	Abcam®	ab204380
ERLIN2	WB: 1/500, IF: 1/50	Cell Signaling Technology®	2959
GM130	IF: 1/100	BD Transduction Laboratories™	610823
MFN2	WB: 1/500	Sigma-Aldrich®	M6444
MFN2	IF: 1/50	Invitrogen	MA5-27647
OPA1	WB: 1/500	BD Transduction Laboratories™	612607
PDI	WB: 1/1000	Abcam®	ab2792
VDAC1	IHC: 1/100	Millipore®	abMAB10527
VDAC1	WB: 1/1000	Abcam®	ab15895
ɣ-Tubulin	WB: 1/10,000	Sigma-Aldrich®	T6557

Supplemental Table 5. Primers specifications.

Designed	Forward	Reverse	Source
SREBF1a	5’-TCAGCGAGGCGGCTTTGGAGCAG-3’	5’-CATGTCTTCGATGTCGGTCA-3’	Invitrogen
SREBF1c	5’-GGAGGGGTAGGGCCAACGGCCT-3’	5’-CATGTCTTCGAAAGTGCAATCC-3’	Invitrogen
RPLP0	5’-ACGGGTACAAACGAGTCCTG-3’	5’-GCCTTGACCTTTTCAGCAAG-3’	Invitrogen

Standardized	Identifier	Source
DGAT1	Hs00201385_m1	Applied Biosystems
DGAT2	Hs01045913_m1	Applied Biosystems
DGKA	Hs00176278_m1	Applied Biosystems
HMGCR	Hs00168352_m1	Applied Biosystems
HMGCS1	Hs00940429_m1	Applied Biosystems
MVD	Hs00964565_m1	Applied Biosystems
RPLP0	Hs99999902_m1	Applied Biosystems
SQLE	Hs01123768_m1	Applied Biosystems
SREBF2	Hs01081784_m1	Applied Biosystems
